# GFAP and NfL as predictors of disease progression and relapse activity in fingolimod-treated multiple sclerosis

**DOI:** 10.1093/brain/awaf433

**Published:** 2025-11-14

**Authors:** Aleksandra Maleska Maceski, Pascal Benkert, Maximilian Einsiedler, Sabine Schaedelin, Johanna Oechtering, Lester Melie-Garcia, Alessandro Cagol, Riccardo Galbusera, Edoardo Galli, Jannis Müller, Sebastian Finkener, Patrice H Lalive, Marjolaine Uginet, Stefanie Müller, Caroline Pot, Amandine Mathias, Renaud Du Pasquier, Robert Hoepner, Andrew Chan, Giulio Disanto, Chiara Zecca, Marcus D’Souza, Lars G Hemkens, Özgür Yaldizli, Tobias Derfuss, Patrick Roth, Claudio Gobbi, David Brassat, Björn Tackenberg, Rosetta Pedotti, Catarina Raposo, Jorge Oksenberg, Ari J Green, Heinz Wiendl, Klaus Berger, Marco Hermesdorf, Fredrik Piehl, David Conen, Ludwig Kappos, Michael Khalil, Cristina Granziera, Ahmed Abdelhak, David Leppert, Eline A J Willemse, Jens Kuhle, for the Swiss Multiple Sclerosis Cohort (SMSC), Amar Zadic, Juan F Vilchez Gomez, Suvitha Subramaniam, Mauricio Rodriguez, Lilian Demuth, Annette Orleth

**Affiliations:** Multiple Sclerosis Centre and Research Center for Clinical Neuroimmunology and Neuroscience (RC2NB), Neurology, Departments of Biomedicine and Clinical Research, University Hospital and University Basel, Basel 4031, Switzerland; Multiple Sclerosis Centre and Research Center for Clinical Neuroimmunology and Neuroscience (RC2NB), Neurology, Departments of Biomedicine and Clinical Research, University Hospital and University Basel, Basel 4031, Switzerland; Department of Clinical Research, University Hospital and University Basel, Basel 4031, Switzerland; Multiple Sclerosis Centre and Research Center for Clinical Neuroimmunology and Neuroscience (RC2NB), Neurology, Departments of Biomedicine and Clinical Research, University Hospital and University Basel, Basel 4031, Switzerland; Multiple Sclerosis Centre and Research Center for Clinical Neuroimmunology and Neuroscience (RC2NB), Neurology, Departments of Biomedicine and Clinical Research, University Hospital and University Basel, Basel 4031, Switzerland; Department of Clinical Research, University Hospital and University Basel, Basel 4031, Switzerland; Multiple Sclerosis Centre and Research Center for Clinical Neuroimmunology and Neuroscience (RC2NB), Neurology, Departments of Biomedicine and Clinical Research, University Hospital and University Basel, Basel 4031, Switzerland; Multiple Sclerosis Centre and Research Center for Clinical Neuroimmunology and Neuroscience (RC2NB), Neurology, Departments of Biomedicine and Clinical Research, University Hospital and University Basel, Basel 4031, Switzerland; Translational Imaging in Neurology (ThINK) Basel, Department of Biomedical Engineering, Faculty of Medicine, University Hospital and University Basel, Basel 4031, Switzerland; Multiple Sclerosis Centre and Research Center for Clinical Neuroimmunology and Neuroscience (RC2NB), Neurology, Departments of Biomedicine and Clinical Research, University Hospital and University Basel, Basel 4031, Switzerland; Translational Imaging in Neurology (ThINK) Basel, Department of Biomedical Engineering, Faculty of Medicine, University Hospital and University Basel, Basel 4031, Switzerland; Dipartimento di Scienze della Salute, Università Degli Studi di Genova, Genova 16132, Italy; Multiple Sclerosis Centre and Research Center for Clinical Neuroimmunology and Neuroscience (RC2NB), Neurology, Departments of Biomedicine and Clinical Research, University Hospital and University Basel, Basel 4031, Switzerland; Translational Imaging in Neurology (ThINK) Basel, Department of Biomedical Engineering, Faculty of Medicine, University Hospital and University Basel, Basel 4031, Switzerland; Multiple Sclerosis Centre and Research Center for Clinical Neuroimmunology and Neuroscience (RC2NB), Neurology, Departments of Biomedicine and Clinical Research, University Hospital and University Basel, Basel 4031, Switzerland; Multiple Sclerosis Centre and Research Center for Clinical Neuroimmunology and Neuroscience (RC2NB), Neurology, Departments of Biomedicine and Clinical Research, University Hospital and University Basel, Basel 4031, Switzerland; Translational Imaging in Neurology (ThINK) Basel, Department of Biomedical Engineering, Faculty of Medicine, University Hospital and University Basel, Basel 4031, Switzerland; Department of Neurology, Cantonal Hospital Aarau, Aarau 5000, Switzerland; Department of Clinical Neurosciences, Division of Neurology, and Department of Medicine, Translational Biomarker Group, Geneva 1211, Switzerland; Diagnostic Department, Division of Laboratory Medicine, Geneva University Hospital, Geneva 1211, Switzerland; Department of Pathology and Immunology, Faculty of Medicine, University of Geneva, Geneva 1211, Switzerland; Department of Clinical Neurosciences, Division of Neurology, and Department of Medicine, Translational Biomarker Group, Geneva 1211, Switzerland; Department of Neurology, Cantonal Hospital St.Gallen, St. Gallen 9007, Switzerland; Service of Neurology and Laboratories of Neuroimmunology, Department of Clinical Neurosciences, Lausanne University Hospital (CHUV) and University of Lausanne, Lausanne 1011, Switzerland; Service of Neurology and Laboratories of Neuroimmunology, Department of Clinical Neurosciences, Lausanne University Hospital (CHUV) and University of Lausanne, Lausanne 1011, Switzerland; Service of Neurology and Laboratories of Neuroimmunology, Department of Clinical Neurosciences, Lausanne University Hospital (CHUV) and University of Lausanne, Lausanne 1011, Switzerland; Department of Neurology, Inselspital, Bern University Hospital, University of Bern, Bern 3010, Switzerland; Department of Neurology, Inselspital, Bern University Hospital, University of Bern, Bern 3010, Switzerland; Multiple Sclerosis Center, Neurocenter of Southern Switzerland, Ente Ospedaliero Cantonale, Lugano 6900, Switzerland; Multiple Sclerosis Center, Neurocenter of Southern Switzerland, Ente Ospedaliero Cantonale, Lugano 6900, Switzerland; Faculty of Biomedical Sciences, Università Della Svizzera Italiana (USI), Lugano 6900, Switzerland; Multiple Sclerosis Centre and Research Center for Clinical Neuroimmunology and Neuroscience (RC2NB), Neurology, Departments of Biomedicine and Clinical Research, University Hospital and University Basel, Basel 4031, Switzerland; Multiple Sclerosis Centre and Research Center for Clinical Neuroimmunology and Neuroscience (RC2NB), Neurology, Departments of Biomedicine and Clinical Research, University Hospital and University Basel, Basel 4031, Switzerland; Multiple Sclerosis Centre and Research Center for Clinical Neuroimmunology and Neuroscience (RC2NB), Neurology, Departments of Biomedicine and Clinical Research, University Hospital and University Basel, Basel 4031, Switzerland; Multiple Sclerosis Centre and Research Center for Clinical Neuroimmunology and Neuroscience (RC2NB), Neurology, Departments of Biomedicine and Clinical Research, University Hospital and University Basel, Basel 4031, Switzerland; Department of Neurology and Clinical Neuroscience Center, University Hospital Zurich and University of Zurich, Zurich 8091, Switzerland; Multiple Sclerosis Center, Neurocenter of Southern Switzerland, Ente Ospedaliero Cantonale, Lugano 6900, Switzerland; Faculty of Biomedical Sciences, Università Della Svizzera Italiana (USI), Lugano 6900, Switzerland; Novartis Pharma AG, Basel 4056, Switzerland; Department of Neurology, Hôpital de la Pitié-Salpêtrière, AP-HP, Paris 75651, France; Department of Neurology, Philipps-University, Marburg 35037, Germany; F.Hoffmann-La Roche Ltd, Basel 4070, Switzerland; F.Hoffmann-La Roche Ltd, Basel 4070, Switzerland; F.Hoffmann-La Roche Ltd, Basel 4070, Switzerland; Department of Neurology and Weill Institute for Neurosciences, University of California, SanFrancisco, CA 94143, USA; Department of Neurology, University of California SanFrancisco, San Francisco, CA 94143, USA; Department of Neurology and Weill Institute for Neurosciences, University of California, SanFrancisco, CA 94143, USA; Department of Neurology, University of California SanFrancisco, San Francisco, CA 94143, USA; Department of Neurology and Neurophysiology, University Medical Center, Freiburg 79106, Germany; Institute of Epidemiology and Social Medicine, University of Münster, Münster 48149, Germany; Institute of Epidemiology and Social Medicine, University of Münster, Münster 48149, Germany; Department of Clinical Neuroscience, Karolinska Institutet, Center for Molecular Medicine, Karolinska University Hospital, Stockholm 17176, Sweden; Center for Neurology, Academic Specialist Center, Stockholm Health Services, Stockholm 17177, Sweden; Population Health Research Institute, McMaster University, Hamilton, Canada L8L2X2; Multiple Sclerosis Centre and Research Center for Clinical Neuroimmunology and Neuroscience (RC2NB), Neurology, Departments of Biomedicine and Clinical Research, University Hospital and University Basel, Basel 4031, Switzerland; Translational Imaging in Neurology (ThINK) Basel, Department of Biomedical Engineering, Faculty of Medicine, University Hospital and University Basel, Basel 4031, Switzerland; Department of Neurology, Medical University of Graz, Graz 8036, Austria; Multiple Sclerosis Centre and Research Center for Clinical Neuroimmunology and Neuroscience (RC2NB), Neurology, Departments of Biomedicine and Clinical Research, University Hospital and University Basel, Basel 4031, Switzerland; Translational Imaging in Neurology (ThINK) Basel, Department of Biomedical Engineering, Faculty of Medicine, University Hospital and University Basel, Basel 4031, Switzerland; Department of Neurology and Weill Institute for Neurosciences, University of California, SanFrancisco, CA 94143, USA; Department of Neurology, University of California SanFrancisco, San Francisco, CA 94143, USA; Multiple Sclerosis Centre and Research Center for Clinical Neuroimmunology and Neuroscience (RC2NB), Neurology, Departments of Biomedicine and Clinical Research, University Hospital and University Basel, Basel 4031, Switzerland; Multiple Sclerosis Centre and Research Center for Clinical Neuroimmunology and Neuroscience (RC2NB), Neurology, Departments of Biomedicine and Clinical Research, University Hospital and University Basel, Basel 4031, Switzerland; Multiple Sclerosis Centre and Research Center for Clinical Neuroimmunology and Neuroscience (RC2NB), Neurology, Departments of Biomedicine and Clinical Research, University Hospital and University Basel, Basel 4031, Switzerland

**Keywords:** Biomarker, translational research, GFAP reference database, progression independent of relapse activity (PIRA)

## Abstract

In multiple sclerosis (MS) patients under therapy, the increase of serum glial fibrillary acidic protein (sGFAP) concentrations is associated with the course of ‘progression in absence of relapse’ (PIRA). While serum neurofilament light chain (sNfL) reflects both response as well as insufficient or lack of efficiency of disease-modifying therapies (DMT), the longitudinal course of sGFAP levels as a drug response marker for future PIRA in relation to specific types of DMT is less clear.

We aimed to compare the predictive capacity of sGFAP and sNfL for PIRA and relapse activity and the longitudinal course in people with MS (PwMS) treated with fingolimod, based on *Z* scores derived from normative values. Overall, 420 PwMS under fingolimod treatment with follow-up of 9.1 years (interquartile range: 7.0–11.0) from the Swiss MS Cohort, contributing 2935 longitudinal serum samples, were included. A reference data set for sGFAP established from 4297 healthy controls across three European and North American cohorts was used to calculate *Z* scores. The longitudinal course and the predictive capacity of biomarkers for time to PIRA and relapse were assessed by Cox proportional hazards and linear mixed-effects models.

In controls, sGFAP concentrations were 13.6% higher in females than males and increased exponentially with age. Altogether, 31.0% of PwMS experienced ≥1 PIRA event. Elevated sGFAP *Z* scores (>0.75) were associated with increased risk of PIRA [hazard ratio (HR): 1.64; 95% confidence interval (CI): 1.16–2.32; *P* = 0.006], while this was not the case for sNfL. Conversely, elevated sNfL predicted relapses (HR: 1.58; 95% CI: 1.13–2.23; *P* = 0.008), while sGFAP did not. Both biomarkers decreased under treatment: sGFAP by 0.19 *Z* score units (ZSU)/10 years (95% CI: −0.27 to −0.11; *P* < 0.001) and sNfL by 0.16 ZSU/10 years (95% CI: −0.27 to −0.06; *P* = 0.002). Serum GFAP remained elevated in PwMS with future PIRA events (estimate: 0.29; 95% CI: 0.07–0.50; *P* = 0.009); no such association was found for sNfL. Serum GFAP and sNfL *Z* scores provide complementary predictive capacity for PIRA and relapse risk. The decrease of sGFAP under fingolimod is a feature not observed with other types of DMT and may hint to a specific anti-neurodegenerative effect of Sphingosine-1-phosphate-receptor modulators on astrocytes.

## Teaser

Maleska Maceski *et al.* analysed serum GFAP and NfL Z scores in patients with MS treated with fingolimod, derived from >4000 healthy controls. Elevated GFAP *Z* scores were associated with a higher risk of progression independent of relapse, while elevated NfL Z scores predicted relapses. Both biomarkers declined over time with treatment.

## Introduction

Multiple sclerosis (MS) is an autoimmune disorder of the CNS characterized by intermittent inflammatory lesion formation and chronic neurodegeneration that manifests clinically as relapses and sustained disability accumulation, respectively. They have been recognized as partly independent pathomechanisms, as the near complete suppression of acute lesion formation by B-cell depleting therapies (BCDT) has only minimal impact on disability accumulation in the absence of relapse-associated worsening (RAW)^1-3^ and hence has coined the term ‘progression independent of relapse activity’ (PIRA).^1,4^

PIRA is present from disease onset and the primary driver of disability accumulation in people with MS (PwMS). Current disease-modifying therapies for MS have failed to halt PIRA, which is now the biggest unmet therapeutic need in MS.^5-9^

Serum neurofilament light chain (sNfL) is a cytoskeletal intermediate filament that occurs exclusively in neurons; its release into biofluids is hence a specific sign of neuronal injury across neurological diseases.^10^ In the context of MS, NfL has been established in recent years as a biomarker of acute disease activity, i.e. relapses and lesion formation in MRI, and for monitoring drug response across all disease-modifying therapies.^11-14^ On its own, the correlation of sNfL with clinical features of progression is however limited,^15-17^ but is strong in combination with serum levels of glial fibrillary acidic protein (GFAP).^18,19^ GFAP is the intermediate filament equivalent of NfL in astrocytes; its increase in body fluids may reflect two different features: in traumatic brain injury and in neuromyelitis optica, GFAP increase results from acute astrocyte damage. In MS, however, GFAP levels reflect inflammatory astrocyte activation, eventually leading to astrogliotic scar formation. Accordingly, several studies have validated the association of serum GFAP (sGFAP) as a biomarker of PIRA,^15,16,18-24^ and total brain and grey matter volume loss as its morphological substrate in MS.^18,25^ Both in relapsing and progressive forms of MS, elevated sGFAP *Z* score levels were predictive for the treatment effect on PIRA of BCDT.^16,18^ Unlike sNfL, most studies on groups of DMTs^15,19,20,23,26^ or specific types of DMTs (BCDT,^16,18^ natalizumab^27-29^) have not observed a longitudinal decrease of GFAP levels in blood or CSF. Little is known about the effect of Sphingosine-1-phosphate-receptor modulators (S1PR-m): only one study in a secondary progressive MS trial (EXPAND^30^) has delivered preliminary results for a reduction of GFAP levels under treatment where siponimod lowered plasma levels of GFAP (pGFAP), and absolute values were associated with reduced measures of disability accumulation.^31^ Furthermore, the lack of increase of pGFAP from baseline to Month 3 under siponimod treatment was predictive for lower risk of disability worsening by end of study (24–36 months) compared to placebo.^32^

Using a cohort of exclusively fingolimod-treated patients with relapsing MS (RMS), we tested whether the concept of sNfL and sGFAP as discrete biomarkers for the prognostication of relapse activity versus. PIRA is a generalizable pattern, i.e. applicable beyond BCDT, based on *Z* scores derived from a large population of normal controls. Second, by delineating the longitudinal dynamics of sGFAP we aimed at validating the decrease of sGFAP as a specific feature under S1PR-m therapy.

## Materials and methods

### Study population

#### Control persons

The GFAP references database was compiled from three control groups with no documented CNS diseases from two European and one USA-based cohorts, encompassing 4297 individuals across six decades of life (Supplementary material, ‘Methods’ section and Supplementary Table 1). Data collected from the reference population included age, sex, body mass index (BMI), estimated glomerular filtration rate (eGFR) and presence of diabetes mellitus.

#### People with MS

For the current study, we utilized prospectively collected data from participants in the Swiss MS Cohort (SMSC; NCT02433028)^33-35^ fulfilling the following inclusion criteria: initiation of fingolimod treatment after, or at most 2 years before, inclusion in the SMSC and a minimum follow-up of at least three 6- or 12-monthly visits. In the ongoing SMSC, demographic, clinical and neuroimaging data are collected every 6 or 12 months alongside blood samples stored at −80°C following standardized procedures.^36^ Standardized clinical assessments with Neurostatus-Expanded Disability Status Scale (EDSS) score calculations are performed by Neurostatus-eTest certified raters at every patient visit.^37,38^ Relapses were defined as new, worsening or recurrent neurologic symptoms that lasted for at least 24 h without fever, infection or adverse reaction to a prescribed medication. Confirmed disability worsening was defined as: an increase in EDSS score of ≥1.5 points from an EDSS score of 0; ≥1.0 point from an EDSS score of 1.0–5; or ≥0.5 points from an EDSS score ≥5.5, confirmed at a subsequent visit at least 6 months apart. Progression independent of relapse activity (PIRA) was defined as confirmed disability worsening without relapses between the reference and confirmation visit.^4^ MRI procedures are described in the Supplementary material.

Institutional review boards at the respective SMSC centres approved this study (BASEC ID PB_2016-01171/EKNZ48/12). Written informed consent was obtained from all participants.

#### Serum GFAP and NfL measurements

Serum GFAP and NfL were measured using the single molecule array (SIMOA) Neurology 2-plex B advantage assay (two-step assay with four-times dilution protocol; Quanterix) on the HDx platform, in accordance with the manufacturer’s instructions, between January 2023 and July 2024. Serum samples (25 µl) were diluted ‘onboard’ 1:4 with sample diluent (75 µl) per determination, while calibrators were measured neat. The diluted sample, 25 µl of paramagnetic beads coated with capture antibodies labelled with two different fluorescent dyes for GFAP and NfL, and 20 µl of biotinylated detector antibodies, were incubated for 35 min 15 s (47 cadences; one cadence is 45 s). Following a wash, 100 µl of streptavidin-conjugated β-galactosidase (Quanterix) were added, followed by a 5 min 15 s (7 cadences) incubation and a wash. Prior to reading, 50 µl of resorufin β-D-galactopyranoside substrate (Quanterix) was added. GFAP and NfL concentrations (pg/ml) were calculated from the calibration curve. Each sample was measured in duplicate at the MS centre at the University Hospital Basel, and the few samples that exceeded an intra-assay coefficient of variation of 20% were reanalysed. Further methodological details are available in the Supplementary material.

### Statistical analysis

#### Demographic and clinical characteristics

Demographic and clinical characteristics were summarized as counts and percentages for categorical or median and interquartile ranges (IQR) for continuous variables. We used a single sample per control person to avoid within-subject correlation and guided the sample selection per patient by overall maximizing the sample coverage over the entire age range. Detailed modelling procedures are described in the Supplementary material.

### GFAP reference database

The sGFAP reference database was used to examine the association between sGFAP and age, BMI, sex, eGFR and a diagnosis of diabetes. Visual inspection revealed a steeper exponential increase of sGFAP levels among older individuals. To further investigate this, we employed a segmented regression model with log-transformed sGFAP as the dependent variable, adjusted for BMI and sex. This model allowed us to estimate the breakpoint at which the rate of sGFAP increase became steeper, treating it as a parameter of the model.

We used a generalized additive model for location, scale and shape (GAMLSS) to flexibly model distributional parameters and extract percentiles and *Z* scores. See the Supplementary material for a detailed description of how the GAMLSS model was developed. In short, the model was based on a Box–Cox *t*-distribution and adjusted for sex and BMI and included a spline term with three degrees of freedom for age. To improve the model fit, we allowed the standard deviation to increase with age. This final model was then used to calculate sGFAP reference curves accounting for these confounders, and to derive *Z* scores (or percentiles) representing deviations from the mean in the control population.

### Associations with time to PIRA in PwMS

We included PwMS who initiated fingolimod treatment and had at least three documented visits to assess potential PIRA, with a first serum sample (index sample) collected 8–24 months (sensitivity analysis 8–18 months) after treatment initiation.

The association between biomarker *Z* scores at index sample and time to first PIRA or relapse event was investigated using individual Kaplan–Meier analyses with dichotomized biomarker *Z* scores, with time starting from index sample. See Supplementary material for detailed information.

### Longitudinal change in biomarkers and PIRA events

We used linear mixed-effects models to explore longitudinal changes in biomarker levels under fingolimod and their association with MS disease features. These analyses included patients with ≥4 years of follow-up and excluded samples collected after fingolimod discontinuation or switching to alternative treatments; however, including PIRA events after fingolimod discontinuation or switching. sGFAP and sNfL *Z* scores were dependent variables in individual models, with the following terms as predictors: age at fingolimod start; EDSS at fingolimod start; recent relapse (<90 days before sampling); and time under fingolimod in years and a random intercept per patient. In addition, we used a binary variable indicating whether the patient experienced a PIRA event during follow-up. To assess whether biomarker dynamics differed in patients with or without PIRA, we tested for an interaction between PIRA and time after fingolimod start. Statistically significant interactions (log-likelihood test) were included in the final model. See the Supplementary material for detailed information.

### Associations of biomarker levels at baseline and future atrophy

Given that MRI sensitivity models suggested a link between sGFAP levels and cross-sectional grey matter volume (GMV), we further investigated this relationship longitudinally. Specifically, we assessed the temporal association between cortical GMV (as the dependent variable) and time, biomarker *Z* score levels at index samples as well as an interaction term between these two variables. In this model, a significant interaction between the biomarker *Z* score and time indicated an association between biomarker levels one year after treatment start and subsequent cortical GMV atrophy. The analysis was adjusted for total intracranial volume, age at start of fingolimod treatment, sex, disease duration at treatment start, EDSS at treatment initiation and recent relapses. Furthermore, an interaction term between age, treatment start, and time was included to account for age-dependent atrophy rates. The association between biomarker level and atrophy was visualized by plotting the marginal effects.

*P*-values <0.05 were considered statistically significant. Analyses were performed in R version 4.3.1.

## Results

### sGFAP reference database

Demographics and biomarker concentrations for the 4297 control subjects included in the sGFAP reference database are reported in Supplementary Table 1. sGFAP was found to be associated with age, BMI and sex. The age-related increase in sGFAP levels followed an exponential pattern, with a higher rate observed in older individuals. Unlike sNfL, which showed no sex differences, sGFAP levels were 13.6% (95% CI: 10.9%–16.4%) higher in females compared to males, across all age groups (Supplementary Fig. 1).

**Figure 1:**
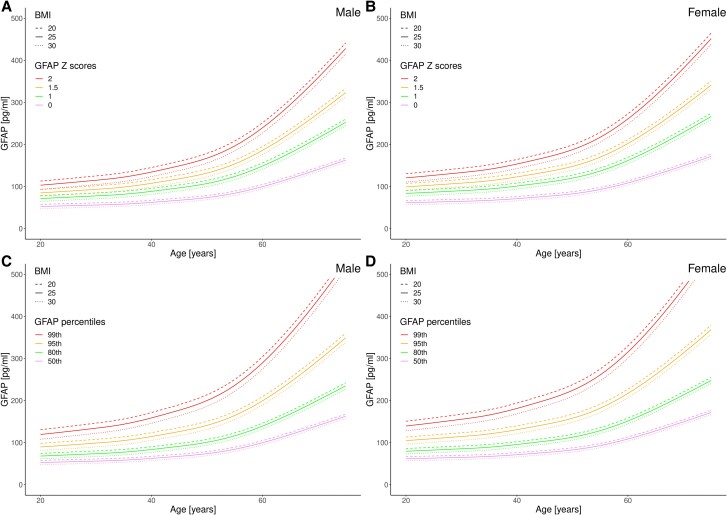
**Serum GFAP *Z* scores and percentile reference curves in control subjects stratified by sex.**  *Z* score (**A** and **B**) and percentile (**C** and **D**) sGFAP reference curves were derived using a generalized additive model for location, scale and shape (GAMLSS), with sGFAP as the dependent variable modelled as a function of age, BMI and sex [males (*left*) and females (*right*)]. *Z* scores express the deviation of an individual’s sGFAP concentration from values in control persons in number of standard deviations, adjusted for confounders (age, BMI and sex). Percentile values (*bottom*) are interchangeable with *Z* scores, where, for example, a *Z* score of 1 corresponds to the 84.1st percentile. BMI = body mass index; sGFAP = serum glial fibrillary acidic protein.

Using a segmented regression model, we determined that sGFAP levels increased by 1.2% per year (95% CI: 1.0%–1.4%) below an estimated age breakpoint of 51.6 (95% CI: 49.7–53.6) years. Beyond this age, the rate rose by an additional 2.6% (95% CI: 2.2%–3.1%) per year, resulting in a total increase of 3.8% annually (Supplementary Fig. 2 and Supplementary Table 2). BMI exhibited a weak but significant inverse relationship with sGFAP, with levels decreasing by 1.4% (95% CI: 1.2%–1.7%) per unit increase of BMI (Supplementary Fig. 1B and Supplementary Table 2). Additionally, reduced renal function, defined by eGFR <60 ml/min/1.73 m², was associated with higher sGFAP *Z* scores, while the presence of diabetes mellitus showed no significant association. The distribution of sGFAP concentrations and calculated reference curves as a function of these three confounders are shown in Fig. 1.

The deviation of an individual’s sGFAP levels from reference values adjusted for confounders is expressed as a *Z* score or percentile value as previously described for NfL.^11,39^ The sGFAP *Z* score calculation tool is publicly available as an online application at https://shiny.dkfbasel.ch/baselgfapreference/ and in a numerical format (Supplementary Tables 3 and 4 and Supplementary Fig. 3).

### Patient characteristics

A total of 420 RMS patients undergoing fingolimod therapy were included in our cohort (Table 1). A relative majority had escalated to fingolimod from platform therapies (32.1%) or were treatment naïve (27.9%). Median follow-up time after the start of fingolimod therapy was 9.1 [interquartile range (IQR): 7.0–11.0] years. During this period, 38.1% of patients experienced a confirmed disability worsening event, mostly PIRA (31.0%). Throughout the follow-up period, 52.4% of PwMS remained on fingolimod, while 27.6% escalated to a higher-efficacy treatment class, and 5.7% switched to another oral DMT.

**Table 1:** Patient characteristics at start of fingolimod therapy, index sample and during follow-up

	Total	Non-PIRA	PIRA	*P* value
**At start of fingolimod**				
Patients	420	290	130	–
Sex, women	272 (64.8)	190 (65.5)	82 (63.1)	0.71
Age, years	40.2 [30.9–47.6]	38.4 [29.4–46.8]	41.7 [33.7–49.2]	**0**.**019**
EDSS	2.0 [1.5–3.0]	2.0 [1.5–3.0]	2.0 [1.5–3.0]	0.40
Disease duration, years	6.4 [1.9–13.0]	6.0 [2.0–12.9]	7.6 [1.8–13.3]	0.31
Previous treatment^a^				0.70
DMT naïve	117 (27.9)	82 (28.3)	35 (26.9)	–
Untreated	64 (15.2)	47 (16.2)	17 (13.1)	–
Platform	135 (32.1)	89 (30.7)	46 (35.4)	–
Orals	26 (6.2)	20 (6.9)	6 (4.6)	–
High efficacy DMT	78 (18.6)	52 (17.9)	26 (20.0)	–
Relapse <3 months before fingolimod start	93 (22.1)	67 (23.1)	26 (20.0)	0.56
Relapse <1 year before fingolimod start	225 (53.6)	152 (52.4)	73 (56.2)	0.55
Relapse in first 2 years after fingolimod start	98 (23.3)	67 (23.1)	31 (23.8)	0.97
CDW event	160 (38.1)	30 (10.3)	130 (100.0)	**<0**.**001**
Follow-up duration after start of fingolimod, years	9.1 [7.0–11.0]	8.6 [6.4–10.5]	10.3 [8.4–11.5]	**<0**.**001**
Stay on fingolimod	220 (52.4)	168 (57.9)	52 (40.0)	**<0**.**001**
Next DMT^b^				
High efficacy DMT	116 (27.6)	68 (23.4)	48 (36.9)	–
Other oral	24 (5.7)	17 (5.9)	7 (5.4)	–
Platform	5 (1.2)	4 (1.4)	1 (0.8)	–
Untreated	47 (11.2)	32 (11.0)	15 (11.5)	–
Follow-up after stop <6 months	8 (1.9)	1 (0.3)	7 (5.4)	–
Time to eventual treatment switch, years	3.2 [1.4–6.0]	2.8 [1.2–4.8]	4.0 [1.9–7.1]	**0**.**007**
**At index sample**				
Treatment start to index sample, years	1.0 [0.9–1.3]	1.0 [0.9–1.3]	1.0 [0.9–1.3]	0.59
sGFAP, pg/ml	75.0 [54.2–102.8]	70.8 [51.4–99.6]	84.2 [59.4–108.8]	**0**.**002**
sGFAP *Z* score	0.2 [−0.7–0.9]	0.0 [−0.9–0.9]	0.5 [−0.5–1.2]	**0**.**003**
sNfL, pg/ml	7.8 [5.5–10.8]	7.6 [5.3–10.6]	7.8 [6.3–11.3]	**0**.**039**
sNfL *Z* score	0.4 [−0.6–1.2]	0.3 [−0.6–1.2]	0.5 [−0.4–1.3]	0.23
**Follow-up**				
Samples	2935	–	–	–
Patients with ≥4 year FU^c^	366	246	120	–
Samples	2743	1797	946	–
Patients with ≥4 year FU, MRI sensitivity model^d^	342	228	114	–
Samples and MRIs	1538	1015	523	–
Patients with ≥4 year FU, atrophy model^e^	308	208	100	–
MRIs	1601	1056	545	–

Variables are expressed as *n* (%) or median [IQR]. *P*-value: comparison between non-PIRA and patients developing PIRA during follow-up (significant findings in bold). CDW = confirmed disability worsening; DMT = disease-modifying treatment; EDSS = Expanded Disability Status Scale; FU = follow-up; IQR = interquartile range; PIRA = progression independent of relapse activity; sGFAP = serum glial fibrillary acidic protein; sNfL = serum neurofilament light chain.

^a^At 6 months before starting fingolimod. Among the 78 people with multiple sclerosis switching treatment from high-efficacy DMTs, 76 switched from natalizumab and 2 from ocrelizumab.

^b^‘High efficacy DMTs’ include: ocrelizumab (*n*: 70), rituximab (*n*: 26), natalizumab (*n*: 14), ofatumumab (*n*: 5), alemtuzumab (*n*: 1); ‘other orals’ include: teriflunomide (*n*: 12), dimethyl fumarate (*n*: 11), cladribine (*n*: 1); ‘Platform’ includes: glatiramer acetate (*n*: 4) and all interferon beta (*n*: 1). Patients having interrupted treatment for >6 months after fingolimod stop were classified as ‘untreated’. Eight patients were followed for less than 6 months after fingolimod cessation.

^c^Fifty-four patients had a follow-up <4 years and were excluded from the longitudinal analysis of biomarker dynamics.

^d^Time points with information on T2-weighted lesion volume, contrast-enhancing lesions, and brain parenchymal fraction available.

^e^Patients having at least two MRI scans during follow-up available.

**Figure 2:**
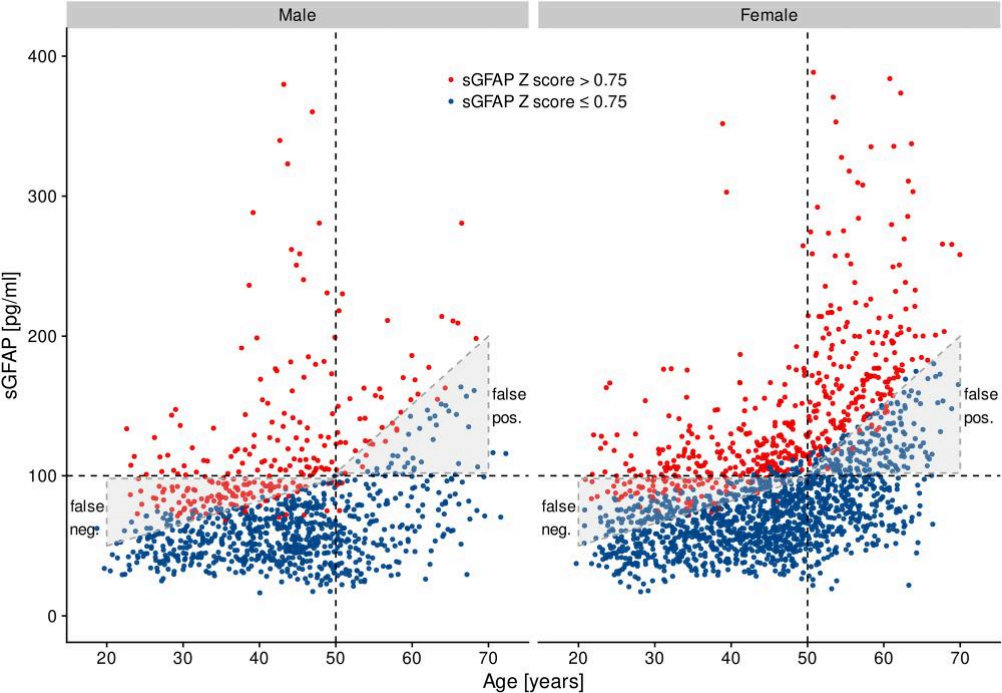
**Serum GFAP *Z* scores in relation to absolute values in people with multiple sclerosis treated with fingolimod.** This scatter plot illustrates the benefit of using *Z* scores instead of absolute biomarker value cut-offs to identify elevated sGFAP levels. Absolute sGFAP concentrations are plotted against age, with data points colour-coded by their *Z* score. Blue filled circles (*Z* score ≤ 0.75) represent non-elevated sGFAP levels relative to the reference population, accounting for physiological factors, while red filled circles (*Z* score > 0.75) indicate elevated sGFAP. An absolute sGFAP cut-off (>100 pg/ml, horizontal dashed line) may lead to misclassifications. False negatives: Younger PwMS (<50 years: vertical dashed line) with elevated sGFAP values (‘high’ *Z* scores) could be missed using an absolute cut-off (red filled circles below the horizontal dashed line). False negatives are more frequent in males (17.2% of 676 samples; Table 2) compared to females (8.9% of 983 samples). False positives: Older PwMS (>50 years) with naturally elevated sGFAP due to ageing may be incorrectly classified as having elevated sGFAP (blue filled circles above the dashed line). Among PwMS >50 years with sGFAP >100 pg/ml, 48.5% (241 of 497 samples) were not elevated by *Z* score (≤0.75), with similar false-positive rates in males (55.2%) and females (46.9%). BMI = body mass index; PwMS = people with multiple sclerosis; sGFAP = serum glial fibrillary acidic protein.

To illustrate the advantage of *Z* scores over absolute biomarker cut-offs, absolute sGFAP concentrations were plotted against age and coloured by sGFAP *Z* score (≤ or > 0.75; Fig. 2 and Table 2). The plot highlights how absolute thresholds can miss elevated levels in younger patients (false negatives) and overestimate them in older patients (false positives), whereas *Z* scores adjust for age-related variation.

**Table 2:** Comparing high absolute sGFAP concentrations with elevated sGFAP *Z* scores and quantification of false negatives and false positives

Sex	Absolute sGFAP (pg/ml)	sGFAP *Z* score	All	Age	
			*n* (%)	≤50 years, *n* (%)	>50 years, *n* (%)
Male	>100	>0.75	144 (73.1)	101 (100)	43 (44.8)
		≤0.75	53 (26.9)	0 (0)	53^b^ (55.2)
		Total	197 (100)	101 (100)	96 (100)
	≤100	>0.75	119 (14.1)	116^a^ (17.2)	3 (1.8)
		≤0.75	726 (85.9)	560 (82.8)	166 (98.2)
		Total	845 (100)	676 (100)	169 (100)
	Total samples		1042 (100)	777 (100)	265 (100)
Female	>100	>0.75	420 (66.9)	207 (91.2)	213 (53.1)
		≤0.75	208 (33.1)	20 (8.8)	188^b^ (46.9)
		Total	628 (100)	227 (100)	401 (100)
	≤100	>0.75	87 (6.9)	87^a^ (8.9)	0 (0)
		≤0.75	1178 (93.1)	896 (91.1)	282 (100)
		Total	1265 (100)	983 (100)	282 (100)
	Total samples		1893 (100)	1210 (100)	683 (100)
Total samples under fingolimod			2935 (100)	1987 (100)	948 (100)

Cross-tabulation of all samples under fingolimod by sex, absolute sGFAP concentration (dichotomized in high versus low based on an arbitrary cut-off of 100 pg/ml), high versus low sGFAP *Z* score (≤ versus >*Z* score 0.75), and age (≤ versus >50 years). Time points differentially classified using absolute sGFAP and *Z* scores are indicated by footnotes. sGFAP = serum glial fibrillary acidic protein.

^a^‘False negatives’: Samples of younger people with multiple sclerosis with ‘low’ absolute sGFAP concentration but elevated *Z* scores, misclassified by using an absolute GFAP cut-off (i.e. red dots below the horizontal line in Fig. 2).

^b^‘False positives’: Samples of older people with multiple sclerosis with ‘high’ absolute sGFAP concentration, which are misclassified as having elevated biomarker levels by using an absolute GFAP cut-off (i.e. blue dots above the horizontal line in Fig. 2).

### Elevated sGFAP levels prognosticated future PIRA and elevated sNfL levels were associated with future relapse activity

**Figure 3:**
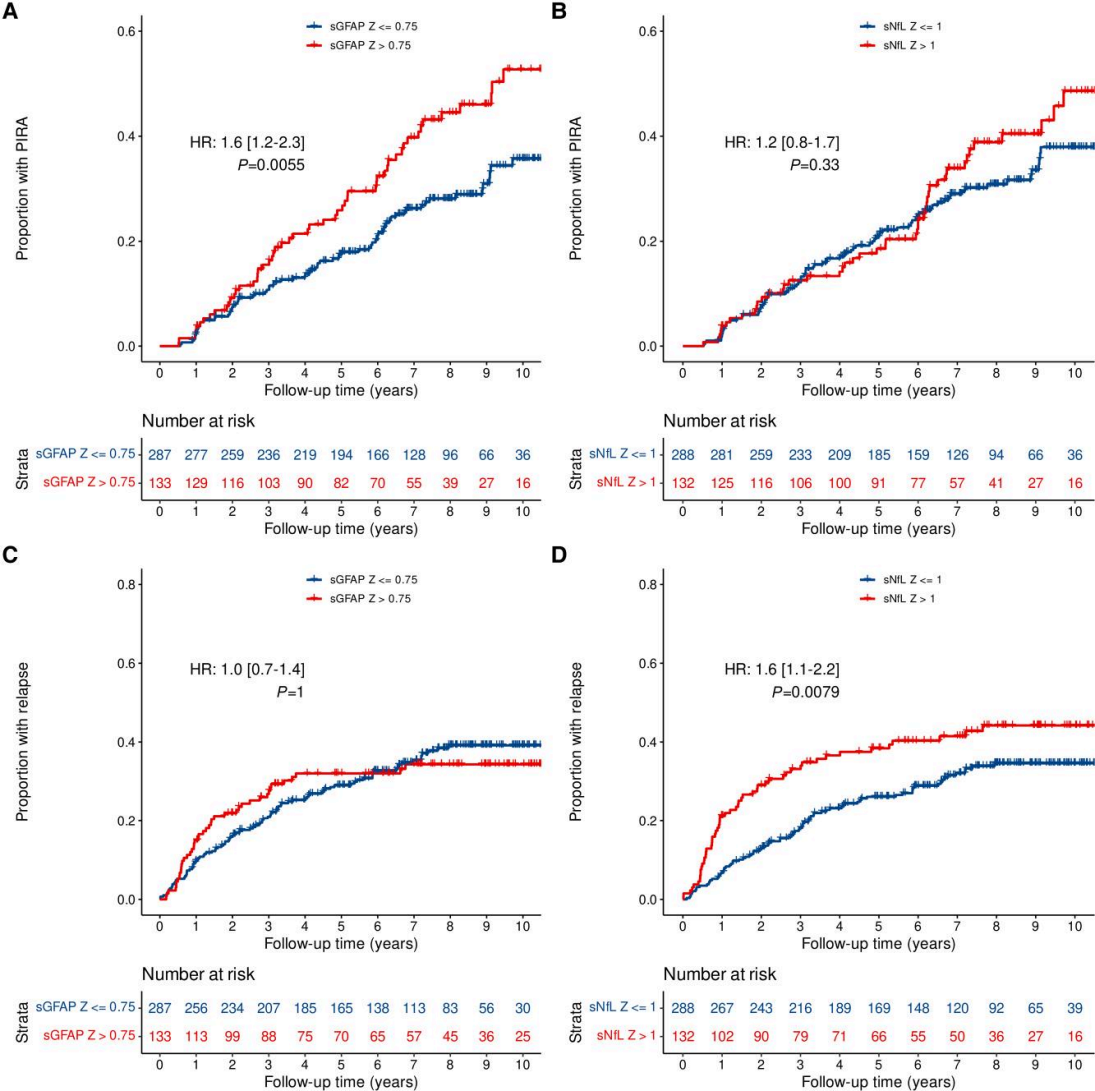
**Elevated sGFAP levels predict future PIRA, and elevated sNfL levels predict relapse activity.** Kaplan–Meier plots showing the proportion of patients experiencing a PIRA (**A** and **B**) or relapse event (**C** and **D**) when presenting with ‘high’ or ‘low’ (*Z* score >0.75 versus ≤0.75) sGFAP levels (**A** and **C**) or ‘high’ versus ‘low’(*Z* score >1 versus ≤1) sNfL levels at index sample. Patients with a high sGFAP *Z* score had a 1.6-fold increased risk of a future PIRA event compared to those with low sGFAP *Z* score (HR: 1.64, 95% CI: 1.16–2.32, *P* = 0.0055; **A**), while sNfL levels were not significantly associated with future PIRA (HR: 1.20, 95% CI: 0.84–1.72, *P* = 0.3251; **B**) Conversely, patients with a high sNfL *Z* score had a 1.6-fold increased risk of subsequent relapse activity compared to those with a low *Z* score (HR:1.58, 95% CI: 1.13–2.23, *P* = 0.0079; **D**), whereas sGFAP levels were not associated with relapse activity (HR:1.00, 95% CI: 0.70–1.43, *P* = 0.9953; **C**). HR = hazard ratio; CI = confidence interval; MS = multiple sclerosis; PIRA = progression independent of relapse activity; sGFAP = serum glial fibrillary acidic protein; sNfL = serum neurofilament light chain; Z = *Z* score.

Patients with a high sGFAP *Z* score (>0.75 versus ≤0.75) at index sample (median 1 year, IQR: 0.9–1.3, after starting fingolimod) had a 1.6-fold increased risk of developing PIRA [hazard ratio (HR): 1.64, 95% CI: 1.16–2.32, *P* = 0.0055], while no significant association was observed for elevated sNfL levels (HR 1.20, 95% CI: 0.84–1.72, *P* = 0.3251) (Fig. 3). These HRs were sustained for sGFAP when alternative *Z* score cut-offs over a wide range were used, but remained unanimously absent for sNfL (Supplementary Fig. 4) and became slightly more pronounced in a multivariable model adjusted for sex, age, EDSS and recent relapse activity (HR: 1.74, 95% CI: 1.22–2.47, *P* = 0.0022; Supplementary Table 5). The greater sensitivity and specificity of sGFAP *Z* scores versus absolute cut-offs enhanced their prognostic value: absolute value sGFAP levels >100 pg/ml were weakly associated with future PIRA events (HR: 1.48, 95% CI: 1.03–2.12, *P* = 0.034; Supplementary Table 6) and lost significance after adjusting for confounders, particularly age. In contrast, the adjusted model using sGFAP *Z* score revealed independent associations of both age (24% increased risk per 10 years of age; *P* = 0.0094) and elevated biomarker levels (69% higher risk when elevated; *P* = 0.0033) with PIRA risk.

While sNfL *Z* scores lacked association with PIRA, they were associated at the index sample with a 1.6-fold increased risk of relapses (*Z* score cut-off: >1 versus ≤1, HR: 1.58, 95% CI: 1.13–2.23, *P* = 0.0079), while this was not the case for sGFAP levels (*Z* score cut-off: >0.75 versus ≤0.75, HR: 1.00, 95% CI: 0.70–1.43, *P* = 0.9953; Fig. 3 and Supplementary Table 7). This pattern remained consistent across a wide range of cut-off values (Supplementary Fig. 4), with relapse risk steadily rising as sNfL *Z* score cut-offs increased. For example, patients with a more pronounced NfL elevation (*Z*score >1.5 versus ≤1.5) were at 2-fold increased relapse risk (HR: 2.00, 95% CI: 1.32–2.92, *P* = 0.0008; Supplementary Fig. 5) with nearly 40% [39.6% (26.7%–50.1%)] experiencing a relapse in the following 2 years, compared to 14% [13.8% (10.1%–17.3%)] of patients with lower sNfL. In the sensitivity analysis, including only samples collected between 8 and 18 months after starting fingolimod treatment, results were very similar (not shown).

### Longitudinal dynamics of sGFAP and sNfL under fingolimod in relation to PIRA

We assessed longitudinal biomarker dynamics in 2743 samples from 366 patients with at least 4 years of follow-up after fingolimod start. Over time, sGFAP levels decreased by 0.19 *Z* score units (ZSU)/10 years (95% CI: −0.27; −0.11, *P* < 0.0001), while sNfL levels decreased by 0.16 ZSU/10 years (95% CI: −0.27; −0.06, *P* = 0.0023).

**Figure 4:**
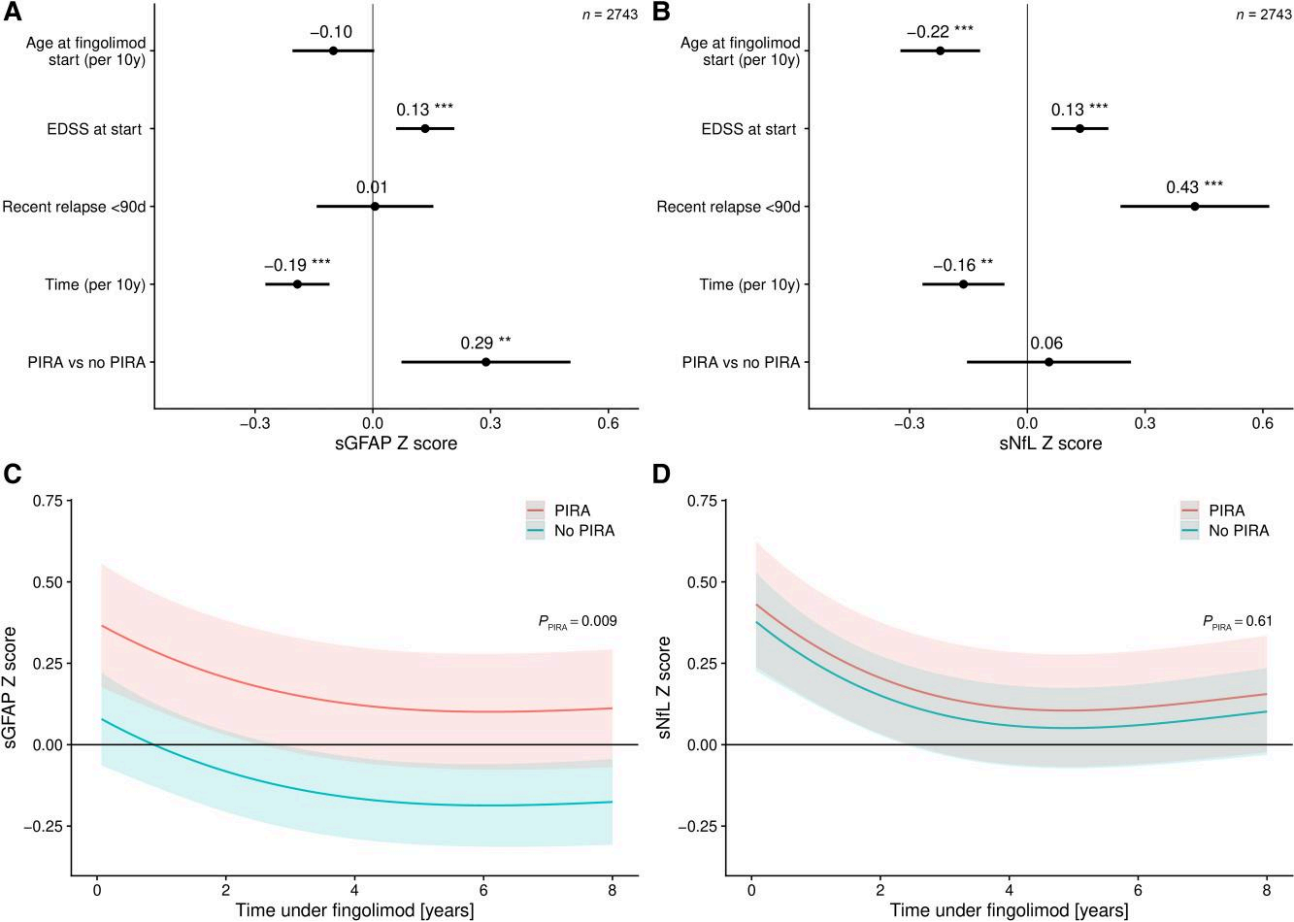
**Longitudinal dynamics of sGFAP and sNfL under fingolimod in relation to PIRA.** (**A**) sGFAP and (**B**) sNfL: estimated effects (filled circles) with 95% CI (error bars) from multivariable mixed-effects models with biomarker *Z* score as dependent variable. (**C**) sGFAP and (**D**) sNfL: marginal effects plots of predicted biomarker *Z* scores along time in relation to PIRA (**A–D** include 2743 samples from 366 patients). A *Z* score of 0 is indicated by a solid line and represents the mean biomarker level in control persons of similar age and BMI and the same sex. Models are adjusted for age, EDSS at fingolimod start and recent relapse <90 days before sampling. Statistical significance indicated as **P < 0.01 or ***P < 0.001. (**A** and **C**) sGFAP *Z* scores decreased by 0.19 *Z* score units per 10 years (*P* < 0.0001) in both PIRA and non-PIRA patients. This decrease was not linear, as visualized in the marginal effects plot with a spline term for time (**C**). The spline model provided a slightly better fit (AIC: 5528) compared to the linear model (AIC: 5542; Supplementary Table 8). Importantly, sGFAP *Z* scores were 0.29 units higher in patients who developed PIRA during follow-up (*P* = 0.0009). (**B** and **D**) Under fingolimod, sNfL decreased by 0.16 *Z* score units per 10 years (*P* = 0.0023), independent of relapse activity (and other covariates), but levels were not associated with PIRA events (*P* = 0.6043; Supplementary Table 8). AIC = Akaike Information Criterion; BMI = body mass index; CI = confidence interval; d = days; EDSS = Expanded Disability Status Scale; FTY = fingolimod; PIRA = progression independent of relapse activity; sGFAP = serum glial fibrillary acidic protein; sNfL = serum neurofilament light chain; y = years.

Patients who experienced a PIRA event during follow-up had higher sGFAP *Z* scores compared to those without PIRA (estimate: 0.29 *Z* scores; 95% CI: 0.07–0.50; *P* = 0.0090; Fig. 4). In contrast, no difference in sNfL *Z* scores was observed between patients with and without PIRA (estimate: 0.06 *Z* scores; 95% CI: −0.15–0.26; *P* = 0.60) (Fig. 4 and Supplementary Table 8).

Higher sGFAP *Z* scores were also associated with higher EDSS scores at fingolimod start, while higher sNfL *Z* scores levels were associated with younger age, higher EDSS scores, and recent relapses.

Sensitivity analyses were conducted on 1538 samples from 342 patients with available MRI data (Supplementary Table 9) and confirmed results of the main analysis: independent of T2-weighted lesion volume, CEL and BPF both sGFAP and sNfL *Z* scores decreased under fingolimod therapy, and higher sGFAP *Z* scores were associated with PIRA events. While sGFAP was associated with BPF, especially cortical GMV (*P* = 0.0031), sNfL was associated with T2-weighted lesion volume and presence of CEL (both *P* < 0.001).

### Association of sGFAP and sNfL levels at index sample with longitudinal cortical grey matter atrophy

**Figure 5:**
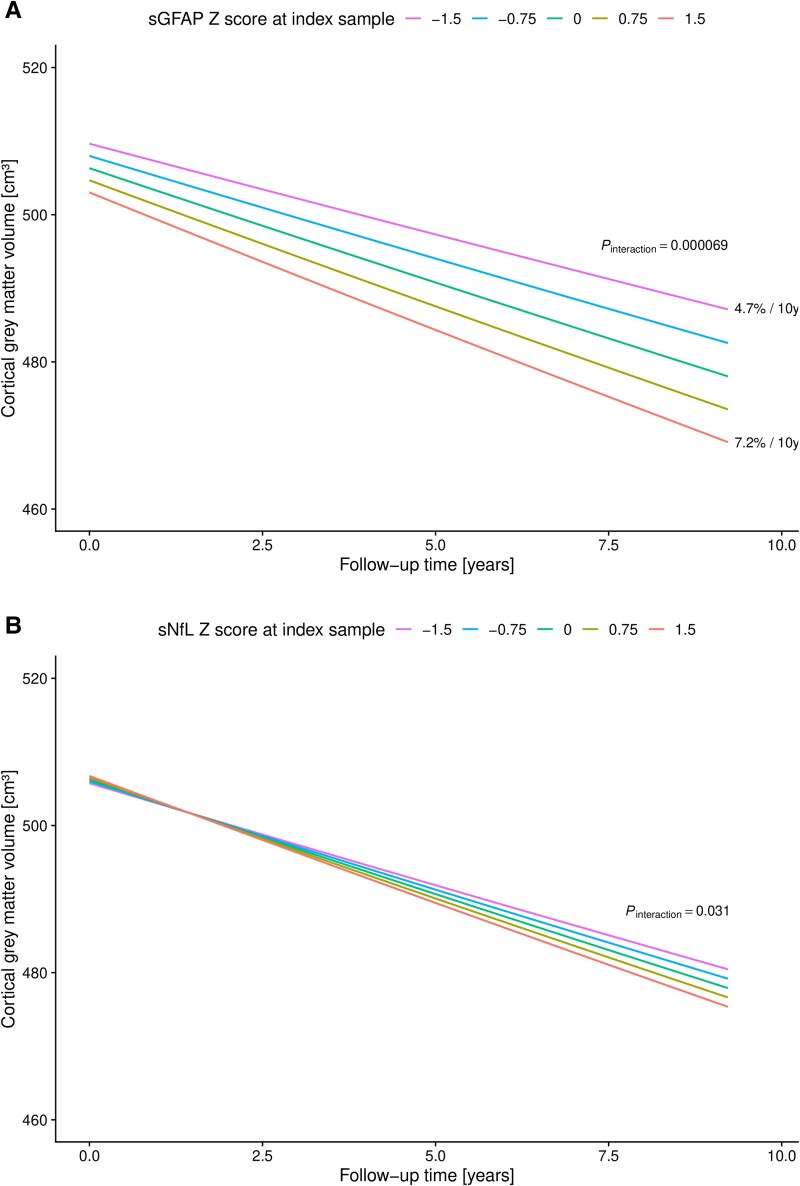
**Association between cortical grey matter atrophy and biomarker levels at 1 year after treatment start.** Association between biomarker levels at index sample and future change in cortical grey matter volume (GMV) over time. In a multivariable mixed effects model with cortical GMV as the dependent variable, this association was described by an interaction term between time after first MRI under fingolimod and biomarker *Z* score as a continuous variable (Supplementary Table 10). The marginal effects were estimated and visualized at five specific *Z* score levels ranging from −1.5 to 1.5 (lines in colour gradient) to give an impression of the magnitude of the effect (308 patients with 1601 MRI examinations). Z scores for 22.4% and 20.8% of patients were between 0.75% and 1.5%, and 7.1% and 14.9% had strongly elevated *Z* scores for sGFAP and sNfL, respectively. (**A**) A one-unit higher sGFAP *Z* score was associated with a 0.89% increase in cortical GMV atrophy over 10 years (*P* < 0.0001). A sGFAP *Z* score of −1.5 was associated with an estimated atrophy rate of 4.7% (95% CI: 3.8–5.6) over 10 years, while a *Z* score of 1.5 was associated with an estimated atrophy rate of 7.2% (95% CI: 6.4–8.0). Atrophy rates are for an age at baseline of 40 years, as we account for age-dependent atrophy rates in the model with an interaction term of baseline age (centred at 40 years) and follow-up time (Supplementary Table 10). (**B**) The sNfL *Z* score increase showed weak association with cortical GMV (*P* = 0.031), which lost significance in the combined biomarker model (Supplementary Table 10; Model 3). CI = confidence interval; sGFAP = serum glial fibrillary acidic protein; sNfL = serum neurofilament light chain; y = years.

Fingolimod-treated patients showed a 6.1% cortical GMV loss over 10 years, which was more pronounced in patients with higher sGFAP levels at index sample (Fig. 5). An increase by one ZSU was associated with an additional 0.89% GMV decrease (estimate: 0.9911; 95% CI: 0.9868–0.9955; *P* < 0.0001); sNfL was only weakly associated with GMV loss (*P* = 0.0309) and lost significance in the combined biomarker model (Supplementary Table 10).

## Discussion

Growing evidence supports the association of increased blood and CSF levels of GFAP with concurrent and future disability accumulation, and with referring MRI measures in MS.^15,16,18-29,31,32,40,41^ However, the translation of this knowledge into application for individual patients has been hindered by the lack of normative reference data that corrects for confounding physiological factors. We have therefore established an expanded normative database that demonstrates that physiologic values of sGFAP are, like sNfL,^11^ strongly dependent on age and BMI, while the 14% higher levels in women across all ages are a feature specific to sGFAP. Absolute value cut-offs to define pathologic levels of sGFAP have the conceptual deficit that they do not compensate for the age-dependent slope of physiologic values, and hence inevitably lead to false-negative or false-positive interpretations of measurements, regardless of the set cut-off value.

The normative values are now accessible as an online tool to interpret individual sGFAP measurements (https://shiny.dkfbasel.ch/baselgfapreference/).

In earlier studies, elevated sGFAP *Z* scores, and less so sNfL *Z* scores, were associated with future PIRA; in turn, sNfL, but not sGFAP, is associated with future relapse activity in MS patients. These studies encompassed MS patients treated with natalizumab^28,29^ and BCDT,^15,16,18^ as well as cohorts under treatment with various additional therapies that were not analysed separately for each compound.^19,20,23,24^ Hence, it was unclear whether the findings were generalisable beyond the two antibody-based high-efficacy therapies.

Current data corroborates the complementary predictive capacity of sNfL and sGFAP as biomarkers of future relapse activity and PIRA, as well as of MRI measures of neurodegeneration^16,18,29^ in a patient cohort uniformly treated with fingolimod. We conclude that the pattern what these two biomarkers reflect is validated and a generalisable feature in the context of MS. Different from other DMTs (rituximab and ocrelizumab,^15,16^ alemtuzumab or natalizumab^29,42^) evaluated for pharmacodynamic effects on GFAP, sGFAP levels decreased under fingolimod therapy over time, potentially reflective of a reduction in absolute terms of biological processes leading to GFAP release. Current findings are consistent with results not published yet in full manuscripts derived from an independent cohort treated with S1PR-m: siponimod in the EXPAND study^30-32^ and more recently fingolimod in a head-to-head comparison with BCDT.^43^

Such associations may have been missed in other studies, because of shorter observation time (≤3 years) and smaller cohort size,^26^ but mainly because the course of GFAP was not analysed in S1PR-m-treated patients separately, but rather in groups of mixed DMTs.^19,24^ This may have precluded delineating potentially specific pharmacodynamic effects of S1PR-m on sGFAP levels as shown in the present results.

The anti-inflammatory effect of S1R-m on T- and B-cells leading to fewer relapses is mediated via the S1PR1, and may largely take place in the ‘periphery’, like for all other DMTs, except for cladribine.^44^ However, S1PR-m cross the blood-brain barrier and accumulate in brain tissue,^45^ and there is ample evidence for a direct pharmacodynamic effect on various cells in the CNS.^44^ Astrocytes, among many other neural cells, express S1PR 1, 2, 3 and 5.^46^ All S1PR-m in clinical use for MS bind to the S1PR1. This receptor may be a major driver for the pharmacodynamic effects of S1PRm on astrocytes, as ponesimod, which binds only to this receptor, led to reduced GFAP expression on astrocytes in an *in vitro* model of neurodegeneration.^47^ All other S1PRms in use for MS bind in addition to receptor 5, for fingolimod this is also the case for S1PR3.^46^ Hence, the modulation of GFAP-related functions in astrocytes could be mediated in addition via these receptors. Other brain-penetrable DMTs, like BTK-inhibitors, need to be investigated to determine whether their PIRA-delaying effect goes along with a reduction of GFAP.

Our study has limitations. First, the normative values for sGFAP are derived from individuals without apparent neurological disease at the time of sample collection. Subclinical neurodegeneration, however, could contribute to elevated sGFAP levels.^48^ Nonetheless, the control cohort's heterogeneity is representative of the general population across a wide age range (20–75 years), which normalizes in part for this factor. However, the accuracy of estimates at more extreme ages may be lower due to the limited number of controls aged below 25 and above 75 years. Second, the study population is predominantly of Caucasian origin, which limits the generalizability of sGFAP *Z* scores to patients of other ethnic backgrounds. Finally, GFAP is known to exist in at least 12 isoforms with unknown quantitative distribution in CSF and blood,^49,50^ which are also captured in current immunoassays. The association of these proteoforms and their breakdown products for the interpretation of GFAP measurements in MS needs to be explored.^50,51^

In conclusion, our findings support the complementary use of sGFAP *Z* scores, alongside sNfL, as a specific measure of neurodegeneration, and, eventually, PIRA in PwMS. The use of normative values that adjust sGFAP measurements for age, BMI and sex is a prerequisite for the accurate interpretation of individual test results in predicting drug response in view of personalized medicine.

## Supplementary Material

awaf433_Supplementary_Data

## Data Availability

Written requests for access to the data reported in this paper will be considered by the corresponding author and a decision made about the appropriateness of the use of the data. If the use is appropriate, a data sharing agreement will be put in place before a fully de-identified version of the dataset used for the analysis with individual participant data is made available. The internet-based application for determination of sGFAP *Z* scores is available under: https://shiny.dkfbasel.ch/baselgfapreference/.
