# Supplementary material for: GFAP and NfL as predictors of disease progression and relapse activity in fingolimod-treated multiple sclerosis
Source: Brain. 2025 Nov 14;149(7):2295–307. doi: 10.1093/brain/awaf433 (PMC13337245; doi:10.1093/brain/awaf433)
Supplement: awaf433_Supplementary_Data [file awaf433_supplementary_data.pdf]

Supplementary material

GFAP and NfL as predictors of disease progression and relapse activity in fingolimod-treated multiple sclerosis

Aleksandra Maleska Maceski†, Pascal Benkert†, et al.

Index:

Page

Supplementary Methods:

1. Control persons 3

2. MRI assessment 4

3. Serum GFAP and NfL measurements 4

4. Establishing the sGFAP Reference Database 5

Supplementary Tables:

Supplementary Table 1. 7
Source of data of persons and samples included in the sGFAP reference database.

Supplementary Table 2. 8
Segmented regression model assessing the association of age, BMI and sex with sGFAP.

Supplementary Table 3. 9
sGFAP reference values at a given age and BMI in females corresponding to a specific Z score (percentile).

Supplementary Table 4. 10
sGFAP reference values at a given age and BMI in males corresponding to a specific Z score (percentile).

Supplementary Table 5.
Univariable and multivariable Cox models assessing the potential of sGFAP and sNfL Z scores at index sample to prognosticate time to PIRA. 11

Supplementary Table 6. 12
Comparison of PIRA prognostication between absolute sGFAP concentrations and sGFAP Z scores.

Supplementary Table 7. 13
Univariable and multivariable Cox models assessing the potential of sGFAP and sNfL Z scores at index sample to prognosticate time to relapse.

Supplementary Table 8. 14
Factors influencing the longitudinal dynamics of biomarker Z scores under fingolimod treatment.

Supplementary Table 9. 15
Sensitivity analyses: clinical and MRI parameters influencing the longitudinal dynamics of biomarker Z scores under fingolimod.

Supplementary Table 10. 16
Association of sGFAP and sNfL Z scores at index sample with future cortical grey matter volume.

Supplementary Figures:

Supplementary Figure 1. 17
Associations between sGFAP and physiological cofounders in control persons.

Supplementary Figure 2. 18
Bi-phasic association between age and sGFAP in control persons approximated by a segmented regression model.

Supplementary Figure 3. 19
Screenshot of the internet-based application providing access to the sGFAP reference database for Z score/percentile calculation.

Supplementary Figure 4. 20
Impact of different sGFAP and sNfL cut-offs on estimated future PIRA and relapse risks.

Supplementary Figure 5. 21
Elevated sNfL under fingolimod therapy predicts future relapse activity.

Supplementary References 22

Supplementary Methods

1. Control persons

Control persons from three cohorts (Supplementary Table 1) were included in the sGFAP reference database (RDB):

1) **Genetic and phenotypic determinants of blood pressure and other cardiovascular risk factors study (GAPP):**

This population-based, prospective cohort study enrolled 2162 healthy adults aged 24–49 years in the Principality of Liechtenstein.[1,2] Persons with cardiovascular disease, diabetes, obstructive sleep apnoea syndrome, daily intake of nonsteroidal anti-inflammatory drugs, a body mass index >35 kg/m² or other major illness or conditions were excluded. All inhabitants of the Principality of Liechtenstein aged 25–41 years were invited to participate. GAPP participants had baseline and one follow-up visit. The institutional review board of the University Hospital Zürich approved the study, and written informed consent was obtained from all participants.

2) **Multiple Sclerosis Expression, Proteomics, Imaging, Clinical Study (EPIC):**

This U.S.-based cohort included 343 healthy adults aged 18–81 years (University of San Francisco).[3] The control consisted of unrelated individuals of MS patients, primarily spouses/partners, friends, and other volunteers (aged 18 to 81 years). A familial history or current diagnosis of MS as well as a relation to another case or control subject or other reported ongoing major illnesses were considered exclusionary for this group. The institutional review boards of University of California San Francisco approved the study, and written informed consent was obtained from all participants.

3) **Establishing the links between subclinical arteriosclerosis and depression (BiDirect):**

BiDirect is observational prospective cohort study[4-6] that was designed to investigate the relationship between depression and (subclinical) arteriosclerosis. Two of three cohorts from BiDirect were used for the generation of the RDB. Participants with depression (n = 922) were recruited randomly from six psychiatric and psychosomatic hospitals in Münster, Germany. Inclusion criteria were (i) age (≥35 and ≤65 years during recruitment) and (ii) current in- or outpatient treatment due to acute depression. Exclusion criteria were (i) compulsory admission, (ii) comorbid dementia, and (iii) comorbid drug abuse (including alcohol). Community dwelling

adults were invited to participate after taking a random sample from the local population register in the city of Münster, Germany (n = 870; age range ≥35 and ≤65 years during recruitment). BiDirect participants who suffered from an episode of depression had comparable sGFAP levels to community dwelling adults (estimate 1.03, CI [0.97-1.10]; p value= 0.3136). BiDirect participants had a baseline and three follow-up visits. The ethics committees of the University of Münster and the Westphalian Chamber of Physicians approved the study, and written informed consent was obtained from all participants. All samples were stored at -80°C until measurement.

2. MRI assessment

Yearly brain MRI scans were performed using the SMSC standardised protocol, which included a 3D Fluid Attenuated Inversion Recovery (FLAIR) and pre-and post-contrast T1 sequences acquired at a spatial resolution of $1mm^3$. Key outcomes included T2-weighted (T2w) lesion volume, number of contrasts enhancing (CEL) T1w lesions, and brain parenchymal fraction (BPF) measured by SAMSEG.[7] Only time points with an available MRI within 3 months from the clinical visit were included.

As recently described,[8] whole brain volume and cerebral cortex volume were measured using SAMSEG following a manual review of the reconstructions. These volumes were normalised by dividing them by the total intracranial volume (TIV) to calculate the brain parenchymal fraction (BPF) and cortical fraction, respectively.

For the MRI sensitivity analysis presented in **Supplementary Table 9**, which examined the relationship between BPF and cortical grey matter with longitudinal biomarker dynamics (Z scores as dependent variables), cross-sectional volumetric measures were utilized. Conversely, for the longitudinal volumetric models assessing biomarker-dependent changes in cortical grey matter volume over time (**Fig. 4, Supplementary Table 10**), longitudinal SAMSEG measurements were used.

3. Serum GFAP and NfL measurements

Three internal calibrator (IC) samples with low sNfL/sGFAP (7.3 and 76.9 pg/ml, respectively), and high concentrations (14.1 and 17.4 pg/ml for sNfL; 147.4 and 128.1 pg/ml for sGFAP) were measured in duplicate in all runs. The sNfL/sGFAP concentrations of these ICs have been assigned during the generation of the sNfL reference database[9] and internal assay validation, respectively and were used for normalisation.[10]

Two internal quality control (QC) serum samples were included in duplicate in each run to assess the inter-assay variability. The mean coefficients of variation (CVs) from these internal QCs were 10.5% and 12.1% for sGFAP (concentrations: 75.6 and 494.4 pg/ml, respectively) and 5.8% and 10.8% for sNfL (concentrations: 8.6 and 104.4 pg/ml, respectively).

4. Establishing the sGFAP Reference Database

4.1. Assessment of relevant comorbidities

A linear mixed-effects model with sGFAP as dependent variable (log-transformed due to the skewed distribution) was used to identify key predictors of sGFAP levels (marginal coefficient of determination of the overall model: R^2=0.31, n=8760 serum samples):

- Age (per year; remaining R^2: 0.04, when excluding this variable)
- BMI (per 1 kg/m^2; remaining R^2: 0.29),
- Sex (remaining R^2: 0.29),
- Binary eGFR <60 ml/min/1.73 m2 (remaining R^2: 0.31),
- Continuous eGFR in those ≥60 ml/min/1.73 m2 (remaining R^2: 0.31)

Based on these analyses (**Supplementary Fig. 1**), BMI, age and sex were in the statistical model to calculate the reference curves. A total 113 samples with eGFR <60 mL/min/1.73 m^2 were excluded as GFAP tended to be increased in presence of kidney dysfunction. Diabetes mellitus was not associated and thus not excluded.

4.2. GAMLSS model development

Reference curves for sGFAP were calculated using the Generalised Additive Models for Location, Scale, and Shape (GAMLSS) framework[11] implemented in the R package gamlss. This approach enabled us to model the relationship between explanatory variables and sGFAP levels while accounting for not only the mean but also the variance, skewness, and kurtosis. We also tested for potential interactions and non-linear association using spline terms.

To develop the model, we extended the free-order procedure introduced by Voncken et al. to handle multiple potential predictor variables.[12] In this optimisation process, each variable, along with all possible interaction terms, was iteratively evaluated using a systematic grid search. For each iteration, a forward and backward stepwise selection was performed for all model parameters. The optimal model was determined based on the Bayesian Information Criterion (BIC), ensuring a balance between model complexity, and fit. This procedure was repeated

iteratively until convergence was achieved after cycling through all potential predictors and interaction terms.

Model performance and fit were evaluated by visually comparing individual data points to the fitted percentiles curves. The normality of the model residuals was evaluated using detrended normal QQ-plots, as described by Buuren & Fredriks and Royston & Wright.[13,14]

4.3. Associations with time to PIRA in PwMS

Cox proportional hazard model analyses were applied with both continuous and dichotomised biomarker Z scores, with sensitivity analyses adjusting for sex, age, EDSS, and recent relapse history. Analyses followed an intention-to-treat approach, with sensitivity analyses restricted to PIRA events occurring under fingolimod, yielding consistent results (data not shown).

Cut-off for dichotomising sNfL Z scores were set at 1.0 (84.1% of controls below this threshold) based on prior work,[15] while for sGFAP a Z score cut-off was set at 0.75 (77.3% of controls below this threshold) to achieve a similar proportion of patients above the cut-off for both biomarkers. Sensitivity analysis with Cox models at various cut-offs validated the robustness of the findings.

4.4. Longitudinal change in biomarkers and PIRA events

We used the Akaike information criterion (AIC) to decide whether a linear or spline term better described the temporal dynamics of the biomarker. Marginal effects of spline models were visually presented, while linear models were used for interpretability, as estimates of remaining parameters differed minimally. We performed a sensitivity analysis in patients with available T2-weighted (T2w) lesion volume, number of contrast-enhancing T1w lesions (CEL), and brain parenchymal fraction (BPF). An alternative model used normalised cortical grey matter volume (GMV) instead of BPF as recent studies showed a stronger association of this substructure with PIRA.[16]

Supplementary Tables

Supplementary Table 1. Source of data of persons and samples included into the sGFAP reference database.

	**Time points: Samples (n)**	**Selected for RDB selected samples[a] (n)**	**Age (years)**	**BMI (kg/m^2)**	**Sex (female, n. %)**
**Genetic and phenotypic determinants of blood pressure and other cardiovascular risk factors study (GAPP)**	BL: 2161<br>FU1: 1534	BL: 1619<br>FU1: 543	34.6 [30.3- 38.8]<br>44.4 [42.7- 45.1]	24.0 [21.8- 26.9]<br>24.4 [22.0- 27.5]	1152 (53.3)
**Multiple Sclerosis Expression- Proteomics-Imaging-Clinical Study (EPIC)**	BL: 343	BL: 343	42.0 [33.0- 51.0]	24.1 [21.7- 27.8]	233 (67.9)
**Establishing the links between subclinical arteriosclerosis and depression (BiDirect)**	BL: 1654<br>FU1: 1226<br>FU2: 934<br>FU3: 908	BL: 544<br>FU1: 258<br>FU2: 239<br>FU3: 751	48.0 [42.3- 54.4]<br>53.5 [47.8- 60.3]<br>56.1 [51.1- 62.6]<br>61.4 [55.0- 66.9]	27.1 [24.2- 31.1]<br>27.8 [24.4- 31.5]<br>27.8 [24.9- 31.6]<br>27.0 [24.2- 30.6]	975 (54.4)
**Total**	8760	**4297**	43.0 [35.5- 53.9]	25.4 [22.6- 28.8]	2360 (54.9)

Legend:

[a]A single sample per control person was selected to avoid adjustment for within-subject correlation. Note that for 1 person from the GAPP study and 138 persons from BiDirect no BL sample was available.

Numbers (%) or medians [IQR] are presented.

Abbreviations: BL: baseline; BMI: body mass index; FU: follow up; IQR: interquartile range; kg/m^2: kilogram per square meter; n: number; RDB: reference database, sGFAP: serum glial fibrillary acidic protein, sNfL: serum neurofilament light chain protein.

Supplementary Table 2. Segmented regression model assessing the association of age, BMI and sex with sGFAP.

	**Estimate**	**CI**	**p-value**
**Age below 51.6 years**	**1.012**	1.010-1.014	**<0.0001**
**Age above 51.6 years**	**1.026**	1.022-1.031	**<0.0001**
**BMI**	**0.986**	0.983-0.988	**<0.0001**
**Gender**	**1.136**	1.109-1.164	**<0.0001**

Legend:

The segmented regression model with log(sGFAP) as dependent variable indicates a bi-phasic association between age and sGFAP. Below 51.6 years (n=3070), sGFAP increases on average by 1.2% per year, while above 51.6 years (n=1227), it increases by additional 2.6% per year. The regression model also shows significant negative associations between sGFAP levels and BMI as well as a gender effect, with females exhibiting on average 13.6% higher sGFAP concentrations compared to males.

Abbreviations: BMI: body mass index; CI: CI: 95% confidence interval; sGFAP: serum glial fibrillary acidic protein.

Supplementary Table 3. sGFAP reference values at a given age and BMI in females corresponding to a specific Z score (percentile).

		Z score (percentiles) in females							
Age	BMI (kg/m^2)	Z=-2 (2.28)	Z=-1 (15.87)	Z=0 (50.00)	Z=1 (84.13)	Z=1.5 (93.32)	Z=2 (97.72)	Z=2.5 (99.40)	Z=3 (99.87)
20	20	34.4	49.1	66.6	90.5	107.4	130.5	164.2	217.7
20	25	31.8	45.4	61.6	83.7	99.3	120.7	151.8	201.3
20	30	29.2	41.7	56.6	76.9	91.2	110.8	139.4	184.9
25	20	34.5	49.8	68.3	93.7	111.9	136.8	173.5	232.3
25	25	31.9	46.2	63.2	86.8	103.6	126.7	160.7	215.2
25	30	29.4	42.5	58.2	79.9	95.4	116.7	148.0	198.1
30	20	34.6	50.6	70.0	97.1	116.6	143.5	183.5	248.1
30	25	32.1	46.9	64.9	90.1	108.2	133.2	170.3	230.3
30	30	29.6	43.3	59.9	83.2	99.8	122.9	157.1	212.5
35	20	34.8	51.6	72.2	101.3	122.4	151.7	195.6	267.2
35	25	32.4	48	67.2	94.2	113.9	141.1	182.0	248.6
35	30	30.0	44.4	62.1	87.2	105.3	130.6	168.4	230.0
40	20	36.0	54	76.3	108.3	131.7	164.4	213.8	295.4
40	25	33.6	50.4	71.3	101.2[a]	123.0	153.6	199.8	275.9
40	30	31.2	46.9	66.3	94.1	114.4	142.8	185.7	256.5
45	20	37.4	56.9	81.4	116.8	143.0	179.9	236.1	329.7
45	25	35.1	53.4	76.3	109.6	134.2	168.8	221.5	309.3
45	30	32.8	49.8	71.3	102.4	125.3	157.7	206.9	289.0
50	20	39.3	60.5	87.7	127.4	157.0	199	263.6	372.4
50	25	37.0	57.1	82.6	120.1	148.0	187.6	248.5	351.0
50	30	34.8	53.6	77.6	112.8	139.0	176.2	233.4	329.7
55	20	42.8	66.9	98.1	144.3	179.1	228.9	306.1	437.7
55	25	40.6	63.5	93.1	136.9	169.9	217.2	290.5	415.3
55	30	38.4	60.0	88.0	129.6	160.8	205.5	274.8	392.8
60	20	48.2	76.5	113.6	169.4	211.7	272.9	368.6	533.5
60	25	46.1	73.1	108.6	161.9	202.4	260.8	352.3	509.9
60	30	44.0	69.8	103.6	154.4	193.0	248.8	336.0	486.3
65	20	54.9	88.4	133.0	200.9	253.1	329	448.9	658.1
65	25	52.8	85.1	128.0	193.3	243.5	316.6	432.0	633.3
65	30	50.7	81.7	122.9	185.8	233.9	304.2	415.0	608.4
70	20	61.9	101.3	154.4	236.6	300.3	393.9	543.3	807.0
70	25	59.9	98.0	149.4	228.9	290.5	381.1	525.6	780.8
70	30	57.9	94.7	144.4	221.2	280.8	368.3	507.9	754.5
75	20	68.7	114.2	176.6	274.4	351	464.8	648.1	976
75	25	66.7	110.9	171.5	266.5	341.1	451.5	629.6	948.2
75	30	64.7	107.7	166.5	258.7	331.1	438.3	611.2	920.5

Legend:
The table provides estimated sGFAP concentrations **in females** corresponding to specific age- and BMI-corrected sGFAP Z score and percentile value as derived from the GAMLSS model calculated on the reference database (https://shiny.dkfbasel.ch/baselgfapreference/).
[a] Reading example: a 40-year-old woman with a BMI of 25 kg/m^2 and sGFAP level of 101.2 pg/ml has an adjusted Z score value of 1 (i.e. the value is 1 standard deviation above the mean value of control persons of same age, BMI and sex). A Z score of 1 corresponds to the 84th percentile, i.e. 84% of the control persons with similar physiological features have lower sGFAP values.
Abbreviations: BMI: body mass index; kg/m^2: kilogram per square meter; sGFAP: serum glial fibrillary acidic protein.

Supplementary Table 4. sGFAP reference values at a given age and BMI in males corresponding to a specific Z score (percentile).

		Z score (percentiles) in males							
**Age**	**BMI (kg/m^2)**	**Z=-2 (2.28)**	**Z=-1 (15.87)**	**Z=0 (50.0)**	**Z=1 (84.13)**	**Z=1.5 (93.32)**	**Z=2 (97.72)**	**Z=2.5 (99.40)**	**Z=3 (99.87)**
20	20	29.8	42.6	57.7	78.5	93.1	113.1	142.3	188.7
20	25	27.2	38.9	52.7	71.7	85.0	103.3	130.0	172.3
20	30	24.6	35.2	47.7	64.8	76.9	93.4	117.6	155.9
25	20	30.0	43.4	59.4	81.6	97.3	119.0	150.9	202.1
25	25	27.5	39.7	54.4	74.7	89.1	108.9	138.2	185.0
25	30	24.9	36.0	49.3	67.8	80.9	98.9	125.4	167.9
30	20	30.2	44.1	61.1	84.8	101.8	125.3	160.2	216.7
30	25	27.7	40.5	56.1	77.8	93.4	115.0	147.0	198.8
30	30	25.2	36.9	51.0	70.8	85.1	104.7	133.9	181.0
35	20	30.6	45.3	63.3	88.8	107.3	133.0	171.5	234.4
35	25	28.1	41.7	58.3	81.8	98.8	122.5	157.9	215.8
35	30	25.7	38.1	53.3	74.7	90.3	111.9	144.3	197.2
40	20	31.8	47.7	67.5	95.7	116.4	145.3	189	261.0
40	25	29.4	44.1	62.4	88.6	107.7	134.5	174.9	241.6
40	30	27.0	40.6	57.4	81.5	99.0	123.7	160.8	222.2
45	20	33.3	50.7	72.5	104.1	127.4	160.3	210.3	293.7
45	25	31.0	47.2	67.5	96.9	118.6	149.2	195.7	273.4
45	30	28.7	43.6	62.5	89.7	109.7	138.1	181.2	253.0
50	20	35.3	54.4	78.8	114.5	141.1	178.9	236.9	334.7
50	25	33.0	50.9	73.8	107.2	132.1	167.5	221.8	313.4
50	30	30.8	47.5	68.7	99.9	123.1	156.1	206.7	292
55	20	38.9	60.8	89.2	131.3	162.9	208.2	278.4	398.1
55	25	36.7	57.4	84.2	123.9	153.7	196.5	262.8	375.7
55	30	34.5	54.0	79.2	116.5	144.6	184.8	247.1	353.2
60	20	44.5	70.5	104.7	156.2	195.2	251.6	339.8	491.8
60	25	42.3	67.2	99.7	148.7	185.8	239.5	323.5	468.2
60	30	40.2	63.8	94.7	141.2	176.5	227.5	307.2	444.6
65	20	51.2	82.5	124.1	187.5	236.2	307.1	419.0	614.2
65	25	49.1	79.2	119.1	179.9	226.6	294.6	402.0	589.4
65	30	47.1	75.8	114.1	172.3	217.1	282.2	385.1	564.5
70	20	58.3	95.5	145.6	223.0	283.0	371.3	512.1	760.7
70	25	56.3	92.2	140.6	215.3	273.3	358.5	494.4	734.4
70	30	54.3	88.9	135.5	207.6	263.5	345.7	476.7	708.2
75	20	65.2	108.4	167.7	260.6	333.4	441.4	615.5	926.9
75	25	63.2	105.2	162.7	252.8	323.4	428.2	597.1	899.2
75	30	61.3	101.9	157.6	245.0	313.4	415.0	578.6	871.4

Legend:
The table provides estimated sGFAP concentrations **in males** corresponding to specific age- and BMI-corrected sGFAP Z score and percentile value as derived from the GAMLSS model calculated on the reference database (https://shiny.dkfbasel.ch/baselgfapreference/).
Abbreviations: BMI: body mass index; kg/m^2: kilogram per square meter; sGFAP: serum glial fibrillary acidic protein.

Supplementary Table 5. Univariable and multivariable Cox models assessing the potential of sGFAP and sNfL Z scores at index sample to prognosticate time to PIRA.

	Endpoint: **Time to PIRA**					
	**sGFAP models**			**sNfL models**		
**Model** (n=420)	**HR**	**CI**	**p-value**	**HR**	**CI**	**p-value**
Univariable<br>Z score > threshold	**1.64**	**1.16-2.32**	**0.0055**	1.20	0.84-1.72	0.3251
Multivariable						
Z score > threshold	**1.74**	1.22-2.47	**0.0022**	1.32	0.91-1.91	0.1406
Woman versus man	0.87	0.61-1.25	0.4571	0.85	0.60-1.22	0.3819
Age (per 1 year)	**1.02**	1.01-1.04	**0.0072**	**1.02**	**1.01-1.04**	**0.0120**
EDSS	0.94	0.82-1.08	0.4070	0.96	0.83-1.11	0.5773
Recent relapse (<90 days)	1.02	0.44-2.35	0.9604	1.09	0.47-2.52	0.8352
Both biomarkers[a]<br>Z score > threshold	**1.61**	**1.13-2.31**	**0.0091**	1.07	0.74-1.55	0.7260
Univariable<br>Z score continuous	**1.23**	**1.06-1.44**	**0.0085**	1.08	0.93-1.25	0.2971
Multivariable						
Z score continuous	**1.26**	**1.07-1.47**	**0.0053**	1.12	0.96-1.30	0.1432
Woman versus man	0.86	0.60-1.23	0.4046	0.85	0.59-1.22	0.3753
Age (per 1 year)	**1.02**	**1.01-1.04**	**0.0107**	1.02	1.01-1.04	0.0118
EDSS	0.95	0.82-1.09	0.4462	0.96	0.83-1.10	0.5364
Recent relapse (<90 days)	1.06	0.46-2.44	0.8950	1.03	0.45-2.37	0.9416
Both biomarkers[a]<br>Z score continuous	**1.23**	**1.04-1.46**	**0.0154**	1.00	0.86-1.17	0.9882

Legend:

Estimates represent hazard ratios and 95% confidence intervals (CIs) from individual univariable and multivariable Cox regression models for time to PIRA. This analysis was performed separately for sGFAP (left columns) and sNfL (right columns) as predictor. In the first 3 models, the biomarker Z score was dichotomised in values above versus below the threshold. The following cut-offs were used: 0.75 Z score for sGFAP and 1.0 Z score for sNfL. In the lower part of the table, biomarker Z scores were used as individual continuous variables. Statistically significant associations are highlighted in bold.

[a] Model with sGFAP and sNfL in the same model.

Abbreviations: CI: 95% confidence interval; EDSS: Expanded Disability Status Scale score; sGFAP: serum glial fibrillary acidic protein; PIRA: progression independent of relapse activity; n: number.

Supplementary Table 6. Comparison of PIRA prognostication between absolute sGFAP concentrations and sGFAP Z scores.

	**sGFAP models**		
**Model** (n=420)	**HR**	**CI**	**p-value**
**Absolute sGFAP concentration**			
sGFAP concentration > vs. ≤100 pg/mL	**1.48**	**1.03-2.12**	**0.0346**
sGFAP concentration > vs. ≤100 pg/mL	1.37	0.93-2.04	0.1142
Woman vs. man	0.86	0.60-1.24	0.4310
Age (per 10 years)	1.15	0.98-1.37	0.0943
BMI (per kg/m^2)	1.02	0.99-1.06	0.1916
**GFAP Z score**			
sGFAP Z score > vs. ≤ 0.75	**1.64**	**1.16-2.32**	**0.0055**
sGFAP Z score > vs. ≤ 0.75	**1.69**	**1.19-2.40**	**0.0033**
Woman vs. man	0.89	0.62-1.28	0.5427
Age (per 10 years)	**1.24**	**1.05-1.45**	**0.0094**
BMI (per kg/m^2)	1.02	0.98-1.06	0.2782

Legend:
Estimates represent hazard ratios and 95% confidence intervals (CIs) from individual univariable and multivariable Cox regression models for time to PIRA. sGFAP values were dichotomised to compare "increased" to "normal" levels. Raw sGFAP > 100 pg/mL was associated with a 48% higher PIRA risk, but this association disappeared after adjusting for sex, age and BMI. In contrast, sGFAP Z scores > 0.75 were associateed with a 64% increased risk, which remained significant – and slightly stronger - after adjustment. The model also revealed an independent effect of age on PIRA (24% higher risk per 10 years), which was masked when using raw values. Statistically significant associations are shown in bold.
Abbreviations: BMI: body mass index; CI: 95% confidence interval; sGFAP: serum glial fibrillary acidic protein; PIRA: progression independent of relapse activity; vs.: versus.

Supplementary Table 7. Univariable and multivariable Cox models assessing the potential of sGFAP and sNfL Z scores at index sample to prognosticate time to relapse.

	Endpoint: **Time to relapse**					
	**sGFAP models**			**sNfL models**		
**Model** (n= 420)	**HR**	**CI**	**p-value**	**HR**	**CI**	**p-value**
Univariable<br>Z score > threshold	1.00	0.70-1.43	0.9953	**1.58**	**1.13-2.23**	**0.0079**
Multivariable						
Z score > threshold	1.01	0.70-1.45	0.9534	**1.61**	**1.14-2.26**	**0.0067**
Woman versus man	1.32	0.92-1.90	0.1305	1.30	0.91-1.87	0.1520
Age (per 1 year)	1.00	0.97-1.02	0.7693	1.00	0.98-1.02	0.8887
EDSS	1.01	0.88-1.16	0.8766	1.00	0.88-1.15	0.9551
Recent relapse (<90 days)	1.80	0.91-3.56	0.0913	1.94	0.98-3.84	0.0584
Both biomarkers*<br>Z score > threshold	0.90	0.62-1.30	0.5658	**1.62**	**1.14-2.30**	**0.0065**
Univariable<br>Z score continuous	1.07	0.92-1.24	0.376	**1.22**	**1.05-1.41**	**0.0074**
Multivariable						
Z score continuous	1.06	0.92-1.23	0.4139	**1.16**	**1.00-1.34**	**0.0497**
Woman versus man	1.37	0.96-1.97	0.0839	1.37	0.96-1.96	0.9086
Age (per 1 year)	**0.96**	**0.95-0.98**	**<0.0001**	**0.97**	**0.95-0.98**	**0.0002**
EDSS	1.12	0.98-1.26	0.0866	1.11	0.98-1.26	0.1091
Recent relapse (<90 days)	1.73	0.87-3.43	0.1196	1.58	0.80-3.14	0.1896
Both biomarkers*<br>Z score continuous	0.99	0.84-1.16	0.9057	**1.22**	**1.05-1.43**	**0.0108**

Legend:
Estimates represent hazard ratios and 95% confidence intervals from individual univariable and multivariable Cox models for time to relapse. This analysis was performed separately for sGFAP (left columns) and sNfL (right columns) as predictor. In the first 3 models, the biomarker Z score was dichotomised in values above versus below the cut-off. The following cutoffs were used: 0.75 Z score for sGFAP and 1.0 Z score for sNfL. In the lower part of the table, biomarker Z scores were used as individual continuous variables. Statistically significant associations are highlighted in bold.
*Model with sGFAP and sNfL in the same model.
Abbreviations: CI: 95% confidence interval; EDSS: Expanded Disability Status Scale score; HR: hazard ratio; n: number; sGFAP: serum glial fibrillary acidic protein; sNfL: serum neurofilament light chain.

Supplementary Table 8. **Factors influencing the longitudinal dynamics of biomarker Z scores under fingolimod treatment.**

	Dependent variable: **sGFAP Z score**			Dependent variable: **sNfL Z score**		
**Model** ($n_{patients}$=366; $n_{samples}$=2743)	**Estimate**	**CI**	**p-value**	**Estimate**	**CI**	**p-value**
Age at fingolimod start (per 10 years)	-0.10	-0.20-0.00	0.0588	**-0.22**	**-0.32--0.12**	**<0.0001**
EDSS at fingolimod start	**0.13**	**0.06-0.20**	**0.0005**	**0.13**	**0.06-0.21**	**0.0003**
Recent relapse (<90 days)	0.01	-0.14-0.15	0.9417	**0.43**	**0.24-0.62**	**<0.0001**
Time (per 10 years)	**-0.19**	**-0.27--0.11**	**<0.0001**	**-0.16**	**-0.27--0.06**	**0.0023**
PIRA versus no PIRA	**0.29**	**0.07-0.50**	**0.0090**	0.06	-0.15-0.26	0.6043

Legend:
Linear mixed-effects models assessing the association between biomarker Z scores as dependent variables - sGFAP (left) and sNfL (right) - with disease characteristics in patients treated with fingolimod. Statistically significant associations are highlighted in bold.
Abbreviations: CI: 95% confidence interval; Time: time after fingolimod start; EDSS: Expanded Disability Status Scale score; sGFAP: serum glial fibrillary acidic protein; sNfL: serum neurofilament light chain; PIRA: progression independent of relapse activity.

Supplementary Table 9. Sensitivity analyses: clinical and MRI parameters influencing the longitudinal dynamics of biomarker Z scores under fingolimod treatment.

($n_{patients}$=342; $n_{samples}$=1538)	Dependent variable: **sGFAP Z score**		
**Model 1**	**Estimate**	**CI**	**p-value**
Age at fingolimod start (per 10 years)	**-0.13**	**-0.24--0.01**	**0.0280**
EDSS at fingolimod start	**0.10**	**0.02-0.18**	**0.0133**
Recent relapse (<90 days)	-0.04	-0.23-0.14	0.6497
Time (per 10 years)	**-0.35**	**-0.50--0.19**	**<0.0001**
PIRA vs no PIRA	**0.30**	**0.08-0.52**	**0.0078**
T2w lesion volume (per doubling)	0.03	-0.01-0.07	0.1612
CEL (yes versus no)	0.15	-0.05-0.35	0.1510
BPF (per 5% decrease)	**0.14**	**0.02-0.25**	**0.0192**
**Model 2**			
Age at fingolimod start (per 10 years)	**-0.16**	**-0.28--0.04**	**0.0089**
EDSS at fingolimod start	**0.10**	**0.02-0.18**	**0.0117**
Recent relapse (<90 days)	-0.05	-0.23-0.14	0.6128
Time (per 10 years)	**-0.39**	**-0.56--0.23**	**<0.0001**
PIRA vs no PIRA	**0.30**	**0.08-0.52**	**0.0076**
T2w lesion volume (per doubling)	0.03	-0.01-0.07	0.1950
CEL (yes versus no)	0.15	-0.06-0.35	0.1594
Cortical nGMV (per 5% decrease)	**0.33**	**0.11-0.54**	**0.0031**
**Model 3**	Dependent variable: **sNfL Z score**		
Age at fingolimod start (per 10 years)	**-0.18**	**-0.29--0.07**	**0.0017**
EDSS at fingolimod start	**0.08**	**0.00-0.16**	**0.0457**
Recent relapse (<90 days)	**0.36**	**0.12-0.60**	**0.0034**
Time (per 10 years)	**-0.26**	**-0.45--0.06**	**0.0097**
PIRA vs no PIRA	0.03	-0.17-0.24	0.7489
T2w lesion volume (per doubling)	**0.09**	**0.04-0.14**	**0.0001**
CEL (yes versus no)	**0.71**	**0.45-0.97**	**<0.0001**
BPF (per 5% decrease)	-0.01	-0.14-0.11	0.8217
**Model 4**			
Age at fingolimod start (per 10 years)	**-0.22**	**-0.33--0.10**	**0.0004**
EDSS at fingolimod start	0.07	-0.00-0.15	0.0653
Recent relapse (<90 days)	**0.35**	**0.11-0.59**	**0.0038**
Time (per 10 years)	**-0.34**	**-0.54--0.14**	**0.0010**
PIRA vs no PIRA	0.03	-0.18-0.24	0.7682
T2w lesion volume (per doubling)	**0.08**	**0.04-0.13**	**0.0004**
CEL (yes versus no)	**0.71**	**0.45-0.97**	**<0.0001**
Cortical nGMV (per 5% decrease)	0.15	-0.10-0.39	0.2466

Legend:
Sensitivity analyses including all longitudinal timepoints under fingolimod with available MRI information. Linear mixed-effects models assessed associations between disease characteristics and biomarker Z scores as dependent variables using two alternative cross-sectional volumetric measures normalised by total intracranial volume: brain parenchymal fraction (BPF) and normalised cortical grey matter volume (GMV). Statistically significant associations are highlighted in bold.
Reading example:
Independent of PIRA and other covariates, sGFAP was associated with BPF, especially normalised cortical GMV (0.33 sGFAP Z score units per 5% decrease in cortical grey matter), while sNfL was not. Only sNfL was associated with T2 weighted lesion volume and the presence of active lesions.
Abbreviations: BPF: brain parenchymal fraction; CEL: T1w contrast enhancing lesions; CI: 95% confidence interval; EDSS: Expanded Disability Status Scale; GMV: grey matter volume; MRI: magnetic resonance imaging; PIRA: progression independent of relapse activity; nGMV: normalised grey matter volume; sGFAP: serum glial fibrillary acidic protein; sNfL: serum neurofilament light chain.

Supplementary Table 10. Association of sGFAP and sNfL Z scores at index sample with future cortical grey matter volume.

	Dependent variable: **Cortical GMV**			
**Interaction term in adjusted model** $n_{patients}$=308; n_{MRIs}=1601	**Estimate**	**% change in 10y and per Z score unit**	**CI**	**p-value**
sGFAP Z score * time (per 10y)	**0.9911**	**-0.89**	**0.9868-0.9955**	**<0.0001**
sNfL Z score * time (per 10y)	**0.9955**	**-0.45**	**0.9913-0.9996**	**0.0309**
**Combined model**				
sGFAP Z score * time (per 10y)	**0.9918**	**-0.82**	**0.9872-0.9965**	**0.0006**
sNfL Z score * time (per 10y)	0.9982	-0.18	0.9938-1.0027	0.4354

Legend:
Multivariable mixed-effects models were constructed with log-transformed cortical grey matter volume (GMV) as the dependent variable to investigate the association between biomarker levels at the index sample and atrophy rates. These associations were modeled using an interaction term between biomarker level and time after first scan under treatment. Separate models analyzed sGFAP Z scores, and sNfL Z score as prognostic factors, and additionally a combined model including both biomarkers was built. All models were corrected for total intracranial volume (TIV; log-transformed), age at fingolimod start, sex, disease duration at fingolimod start, EDSS at fingolimod start, recent relapse and interaction terms with age since first scan to account for age-related atrophy effects. Estimates were back-transformed to represent percentage changes in cortical grey matter volume over 10 years per Z score unit increase. Marginal effects for defined Z score values are shown in **Fig. 4.** Statistically significant associations are highlighted in bold.
Reading example:
A one unit increase in sGFAP Z score was associated with an additional cortical GMV loss of -0.89% over 10 years. sNfL Z scores showed weaker associations with cortical GMV and were not significant in the combined biomarker model.
Abbreviations: CI: 95% confidence interval; GMV: grey matter volume; n:number; sGFAP: serum glial fibrillary acidic protein; sNfL: serum neurofilament light chain.

Supplementary Figure 1. Associations between sGFAP and physiological confounders in control persons.

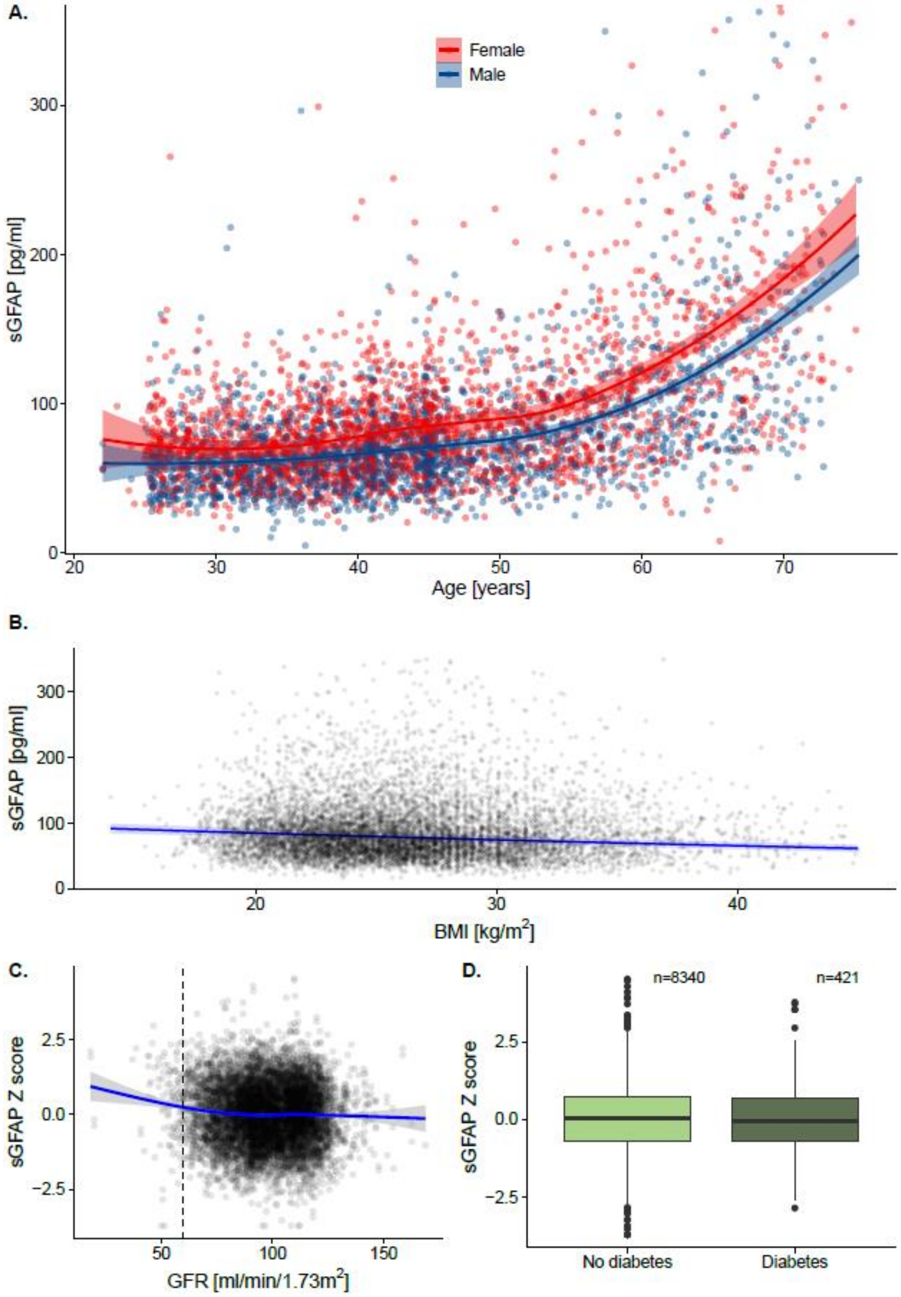

Legend:
Associations of cofounding factors with sGFAP were assessed in all samples (n=8760) of individuals in the reference database.
A.) sGFAP levels increased exponentially with a sharper rise observed after the age of 50. On average, females exhibited 13.6% higher sGFAP levels than males across the entire age range.
B.) sGFAP levels steadily decreased as BMI increased. Note: 35 samples from control individuals with BMI >45 are not shown in the figure.
C.) Kidney dysfunction, as indicated by an estimated Glomerular Filtration Rate (eGFR) <60 ml/min/1.73m^2 (dotted line), was associated with elevated sGFAP levels. As a result, participants with eGFR <60 ml/min/1.73 were excluded from the RDB.
D.) A diagnosis of diabetes mellitus was not associated with sGFAP levels (0.992; 0.936-1.051; p=0.784 in a mixed-effects model adjusted for age, BMI, and sex).
Abbreviations: BMI: body mass index; eGFR: estimated glomerular filtration rate; sGFAP: serum glial fibrillary acidic protein; RDB: reference database.

Supplementary Figure 2. Bi-phasic association between age and sGFAP in control persons approximated by a segmented regression model.

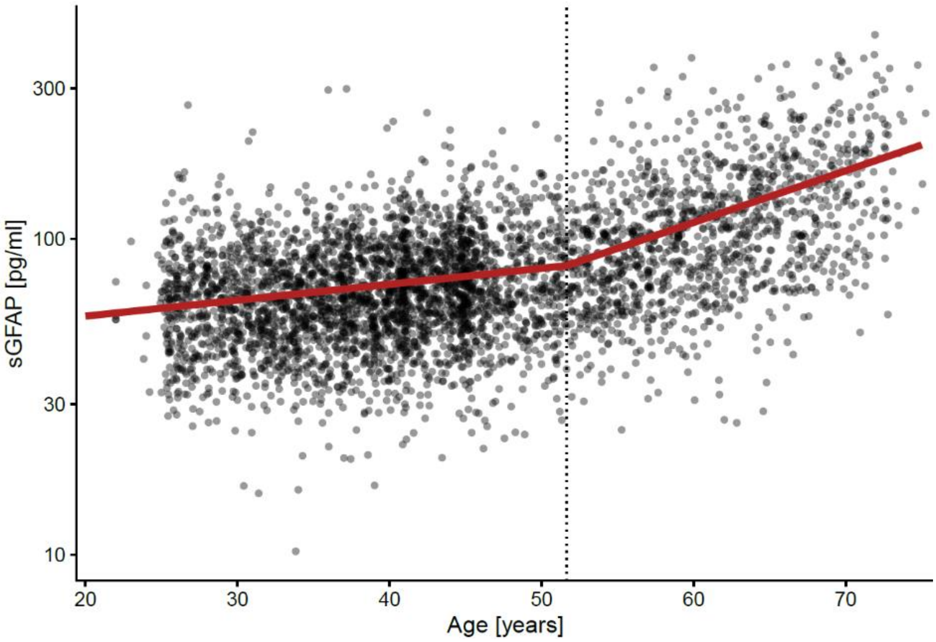

Legend:
The segmented regression model demonstrated that the association between sGFAP and age could be approximated by two distinct linear models with log(sGFAP) as a dependent variable. The model estimated the optimal breakpoint to occur at age 51.6 (+/- 1.0) years. In control persons younger than 51.6 years, sGFAP increased with an estimated 1.2% per year (estimate: 1.012; 95%CI [1.010-1.014]; $p < 0.0001$). For individuals aged 51.6 or older, age was associated with an additional sGFAP increase of 2.6% per year (estimate: 1.026; [1.022-1.031]; $p < 0.0001$) resulting in an overall sGFAP increase of 3.8% per year in this age group. Dots represent individual samples (one per person) and red lines indicate the slopes estimated from the segmented regression model.
Abbreviation: CI: 95% confidence interval; sGFAP: serum glial fibrillary acidic protein.

Supplementary Figure 3. Screenshot of the internet-based application providing access to the sGFAP reference database for Z score/percentile calculation.

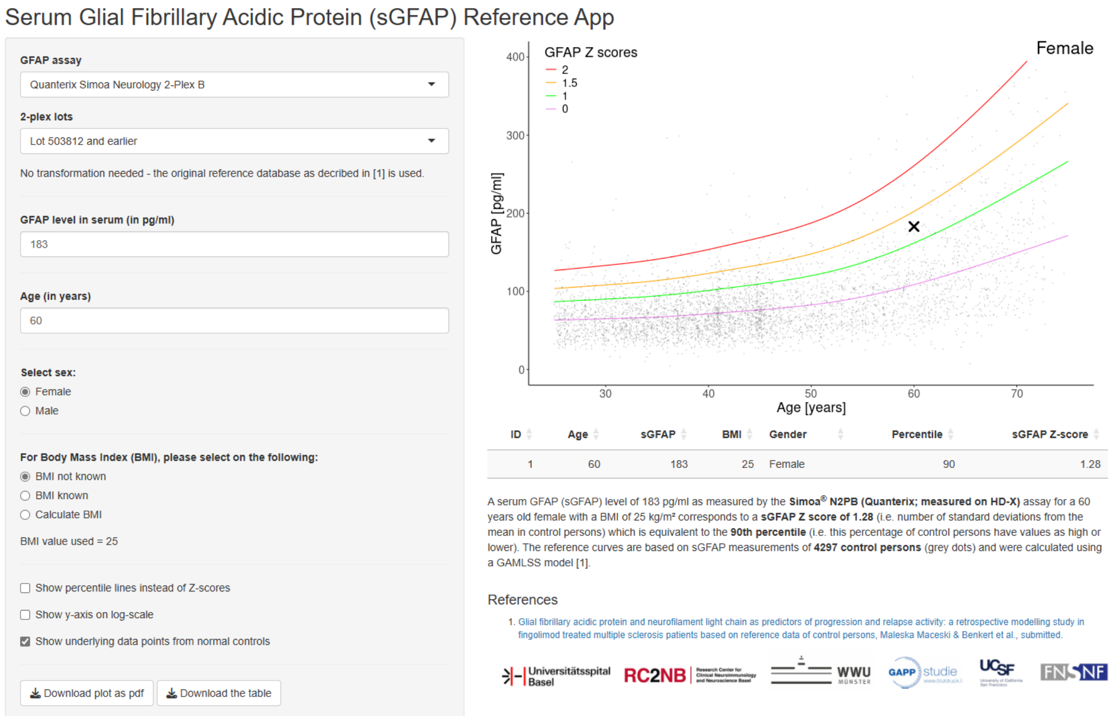

Legend:
Online application for the calculation and visualisation of age-, sex- and BMI-specific sGFAP Z scores and percentiles. The website is accessible under the following address: https://shiny.dkfbasel.ch/baselgfapreference/.
Abbreviations: BMI: body mass index; N2PB: neurology 2 plex assay; sGFAP: serum glial fibrillary acidic protein; Simoa: single molecule array technology.

Supplementary Figure 4. Impact of different sGFAP and sNfL cut-offs on estimated future PIRA and relapse risks.

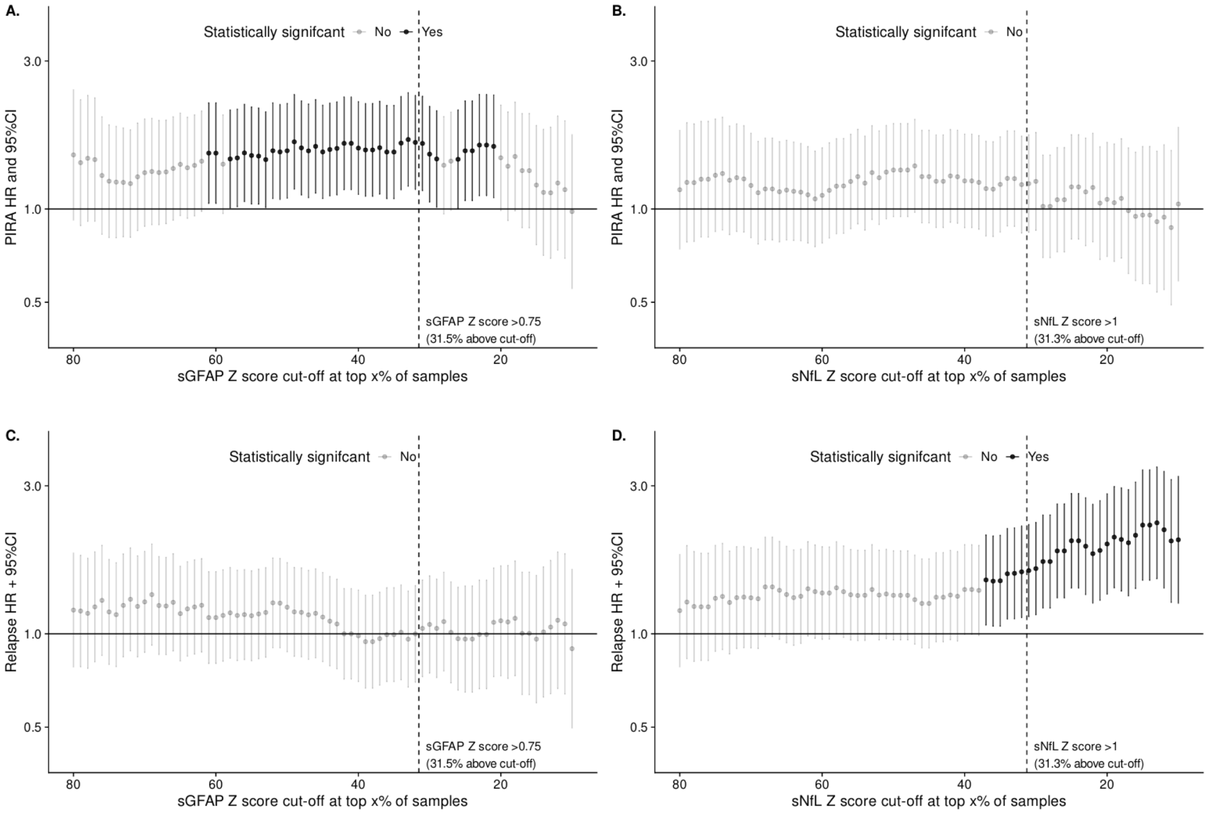

Legend:
Impact of the cut-off value for sGFAP (**A. and C.**) and sNfL Z score (**B. and D.**) on the resulting estimated risk of future PIRA (upper panels) and future relapse activity (lower panels). Hazard ratios (HR; dots) and 95% confidence intervals (CI, vertical lines) derived from individual Cox regression models investigating the association between dichotomised sGFAP Z score and sNfL Z score and time to PIRA and time to relapse are shown. Significant associations are indicated by CIs that exclude 1 and are depicted in black whereas non-significant associations are shown in grey. The cut-offs used in the Kaplan-Meier analysis (**Fig. 2** in the manuscript) are depicted by dashed vertical lines at sGFAP Z score 0.75 and sNfL Z score 1, which classify approximately 30% of the highest values in the cohort as elevated (31.7% with sGFAP Z score >0.75, and 31% with sNfL Z score >1).
Interpretation: For sGFAP, the sensitivity analysis demonstrates that a wide range of cut-off values (ranging from 20% to 60% of the top values) results in significant associations with time to PIRA. For sNfL, the analysis reveals strong associations with relapse activity with hazard ratios increasing as the cut-off values become more stringent.
Abbreviations: HR: hazard ratio; CI: 95% confidence interval; PIRA: progression independent of relapse activity; sGFAP: serum glial fibrillary acidic protein; sNfL: serum neurofilament light chain.

Supplementary Figure 5. Elevated sNfL under fingolimod therapy predicts future relapse activity.

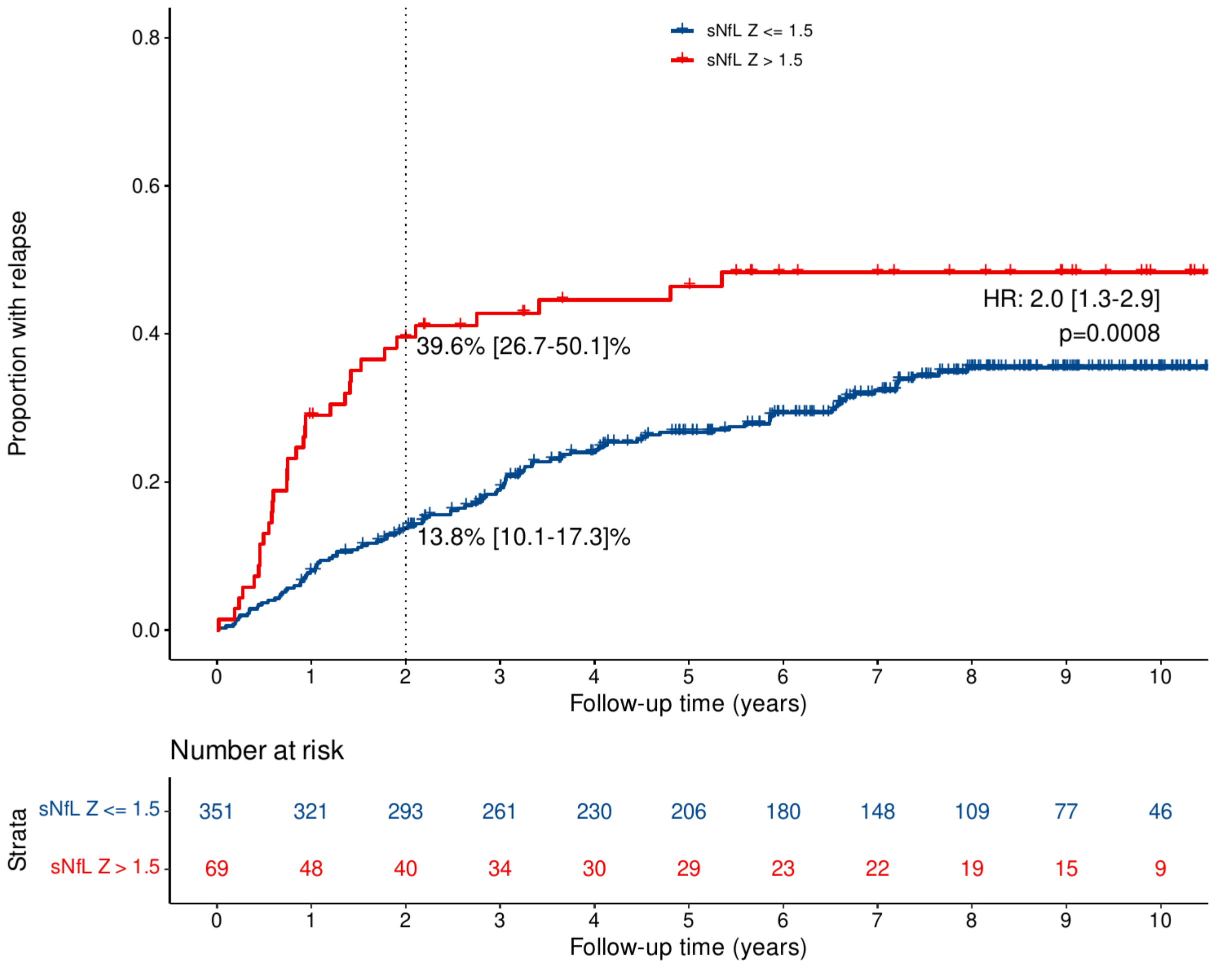

Legend:
Kaplan-Meier plot showing the proportion of patients experiencing a relapse stratified by elevated sNfL levels (Z score >1.5 vs. ≤1.5) at the index sample median 1 year after fingolimod start. Patients with a sNfL Z score >1.5 had a 2-fold increased risk of future relapse, with nearly 40% experiencing a relapse in the following 2 years, compared to 14% of patients with lower sNfL. These findings underline the potential of sNfL as an early marker of treatment non-response in MS.
Abbreviations: HR: hazard ratio; CI: 95% confidence interval; MS: multiple sclerosis; sNfL: serum neurofilament light chain; Z: Z scores.

Supplementary References

1. Conen D, Schön T, Aeschbacher S, *et al.* Genetic and phenotypic determinants of blood pressure and other cardiovascular risk factors (GAPP). *Swiss Med Wkly.*2013;143:w13728.
2. Krisai P, Aeschbacher S, Ruperti Repilado FJ, *et al.* Healthy lifestyle and glucagon-like peptide-1 in young and healthy adults: A population-based study. *Prev Med.*2017;101:72-76.
3. Baranzini SE, Wang J, Gibson RA, *et al.* Genome-wide association analysis of susceptibility and clinical phenotype in multiple sclerosis. *Hum Mol Genet.*2009;18(4):767-78.
4. Teismann H, Wersching H, Nagel M, *et al.* Establishing the bidirectional relationship between depression and subclinical arteriosclerosis--rationale, design, and characteristics of the BiDirect Study. *BMC Psychiatry.*2014;14:174.
5. Teuber A, Sundermann B, Kugel H, *et al.* MR imaging of the brain in large cohort studies: feasibility report of the population- and patient-based BiDirect study. *Eur Radiol.*2017;27(1):231-238.
6. Wersching H, Berger K. [New cohorts. The BiDirect study]. *Bundesgesundheitsblatt Gesundheitsforschung Gesundheitsschutz.*2012;55(6-7):822-3. Neue Kohorten. Die BiDirect-Studie.
7. Puonti O, Iglesias JE, Van Leemput K. Fast and sequence-adaptive whole-brain segmentation using parametric Bayesian modeling. *Neuroimage.*2016;143:235-249.
8. Cagol A, Benkert P, Melie-Garcia L, *et al.* Association of Spinal Cord Atrophy and Brain Paramagnetic Rim Lesions With Progression Independent of Relapse Activity in People With MS. *Neurology.*2024;102(1):e207768.
9. Benkert P, Meier S, Schaedelin S, *et al.* Serum neurofilament light chain for individual prognostication of disease activity in people with multiple sclerosis: a retrospective modelling and validation study. *Lancet Neurol.*2022;21(3):246-257.
10. Simrén J, Andreasson U, Gobom J, *et al.* Establishment of reference values for plasma neurofilament light based on healthy individuals aged 5-90 years. *Brain Commun.*2022;4(4):fcac174.
11. Rigby RA, Stasinopoulos DM. Generalized additive models for location, scale and shape. *Applied Statistics.*2005;54(3):507-554.
12. Voncken L, Albers CJ, Timmerman ME. Improving confidence intervals for normed test scores: Include uncertainty due to sampling variability. *Behav Res Methods.*2019;51(2):826-839.
13. van Buuren S, Fredriks M. Worm plot: a simple diagnostic device for modelling growth reference curves. *Stat Med.*2001;20(8):1259-77.
14. Royston P, Wright EM. Goodness-of-fit statistics for age-specific reference intervals. *Stat Med.*2000;19(21):2943-62.
15. Benkert P, Maleska Maceski A, Schaedelin S, *et al.* Serum Glial Fibrillary Acidic Protein and Neurofilament Light Chain Levels Reflect Different Mechanisms of Disease Progression under B-Cell Depleting Treatment in Multiple Sclerosis. *Ann Neurol.*2024;97(1):104-15.
16. Cagol A, Schaedelin S, Barakovic M, *et al.* Association of Brain Atrophy With Disease Progression Independent of Relapse Activity in Patients With Relapsing Multiple Sclerosis. *JAMA Neurol.*2022;79(7):682-692.
